# Three Chalconoids and a Pterocarpene from the Roots of *Tephrosia aequilata*

**DOI:** 10.3390/molecules22020318

**Published:** 2017-02-20

**Authors:** Yoseph Atilaw, Sandra Duffy, Matthias Heydenreich, Lois Muiva-Mutisya, Vicky M. Avery, Máté Erdélyi, Abiy Yenesew

**Affiliations:** 1 Department of Chemistry, University of Nairobi, P.O. Box 30197, 00100 Nairobi, Kenya; gebreyos@gmail.com (Y.A.); loismwikali@yahoo.com (L.M.-M.); 2 Discovery Biology, Griffith Institute for Drug Discovery, Griffith University, Nathan, QLD 4111, Australia; sandra.duffy@griffith.edu.au (S.D.); v.avery@griffith.edu.au (V.M.A.); 3 Institut für Chemie, Universität Potsdam, Karl-Liebknecht-Str. 24-25, D-14476 Potsdam, Germany; mheydenr@uni-potsdam.de; 4 Department of Chemistry and Molecular Biology, University of Gothenburg, SE-412 96 Gothenburg, Sweden; 5 Swedish NMR Centre, University of Gothenburg, SE-405 30 Gothenburg, Sweden

**Keywords:** *Tephrosia aequilata*, chalcone, retrochalcone, aequichalcone A, aequichalcone B, aequichalcone C, pterocarpene, antiplasmodial

## Abstract

In our search for new antiplasmodial agents, the CH_2_Cl_2_/CH_3_OH (1:1) extract of the roots of *Tephrosia aequilata* was investigated, and observed to cause 100% mortality of the chloroquine-sensitive (3D7) strain of *Plasmodium falciparum* at a 10 mg/mL concentration. From this extract three new chalconoids, *E*-2′,6′-dimethoxy-3′,4′-(2′′,2′′-dimethyl)pyranoretrochalcone (**1**, aequichalcone A), *Z*-2′,6′-dimethoxy-3′,4′-(2′′,2′′-dimethyl)pyranoretrochalcone (**2**, aequichalcone B), 4′′-ethoxy-3′′-hydroxypraecansone B (**3**, aequichalcone C) and a new pterocarpene, 3,4:8,9-dimethylenedioxy-6a,11a-pterocarpene (**4**), along with seven known compounds were isolated. The purified compounds were characterized by NMR spectroscopic and mass spectrometric analyses. Compound **1** slowly converts into **2** in solution, and thus the latter may have been enriched, or formed, during the extraction and separation process. The isomeric compounds **1** and **2** were both observed in the crude extract. Some of the isolated constituents showed good to moderate antiplasmodial activity against the chloroquine-sensitive (3D7) strain of *Plasmodium falciparum*.

## 1. Introduction

*Tephrosia* (family Leguminosae) is a pantropical genus encompassing more than 350 species, 110 of which are found in Africa, and 30 of these in Kenya [1]. Some *Tephrosia* species are traditionally used in herbal medicine, while other members of this genus are known as a fish poison and as insecticides [1,2]. The genus produces chalconoids, flavonoids and isoflavonoids, most of which are substituted with a prenyl or a modified prenyl group [3]. In East Africa, the roots of *Tephrosia aequilata* are used to cure venereal diseases and to reduce pain [4]. Previous phytochemical investigation of the roots of this plant yielded a new pterocarpan, 3,4:8,9-dimethylene- dioxypterocarpan, and four known chalconoids, namely praecansone A, praecansone B, Z-praecansone A and demethylpraecansone B [1]. Some chalconoids such as licochalcone A are known for their in vitro and in vivo antimalarial activities [5]. As *Tephrosia aequilata* was reported to produce chalconoids [1], we chose to investigate this plant. The crude CH_2_Cl_2_/CH_3_OH (1:1) extract of the roots of *T. aequilata* showed antiplasmodial activity in a preliminary assay, and chromatographic separation of this extract led to the isolation of four new compounds: *E*-2′,6′-dimethoxy-3′,4′-(2′′,2′′-dimethyl)pyranoretrochalcone (**1**), *Z*-2′,6′-dimethoxy-3′,4′-(2′′,2′′-di-methyl)-pyranoretrochalcone (**2**), 4′′-ethoxy-3′′-hydroxypraecansone B (**3**), and 3,4:8,9-di-methylene-dioxypterocarpene (**4**), along with seven known compounds (**5**–**11**). The characterization and the antiplasmodial activities of these compounds are discussed here.

## 2. Results and Discussion

**Figure 1:**
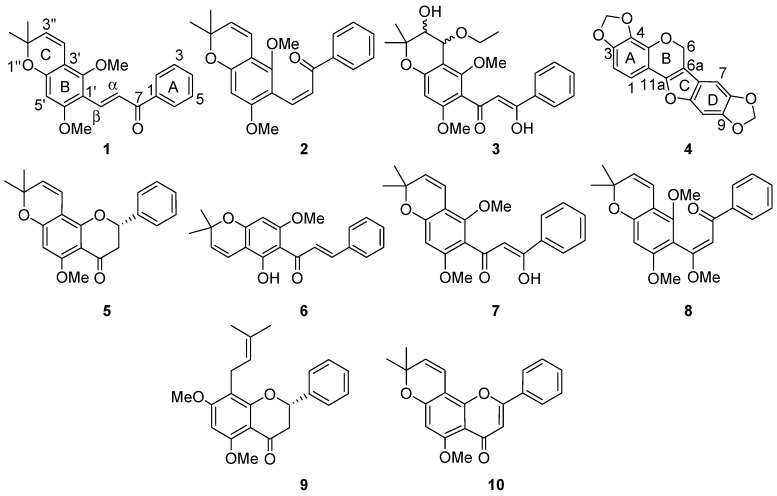
Compounds **1**–**10** isolated from the roots of *Tephrosia aequilata.*

Extraction of the air dried roots of *T. aequilata* with CH_2_Cl_2_/CH_3_OH (1:1) at room temperature, followed by chromatographic separation, afforded 11 compounds. Of these, obovatin methyl ether (**5**) [6,7], obovatachalcone (**6**) [7], praecansone B (**7**) [8], *Z*-praecansone A (**8**) [9], candidone (**9**) [2], isopongaflavone (**10**) [10,11], and β*-*sitostrol-3-*O*-glucoside (**11**) [12] are known, and were identified by comparison of their observed and reported spectroscopic and physical data. Compounds **1**–**4** (Figure 1) are new and were identified by NMR spectroscopic and mass spectrometric analyses.

Compound **1** was isolated as a yellow paste showing UV absorption maxima at 240, 290 and 370 nm, typical of a chalconoid chromophore [13]. Based on HRESIMS analysis ([M + H]^+^ obs *m*/*z* 351.1585, calcd 351.1591), and ^1^H- and ^13^C-NMR spectral data (Table 1), the molecular formula C_22_H_22_O_4_ was assigned. The ^1^H-NMR signals observed at δ_H_ 7.96 (d, *J* = 16.0 Hz) and δ_H_ 8.15 (d, *J* = 16.0 Hz) correspond to the H-α and H-β, respectively, of a chalconoid skeleton possessing *E*-geometry. The corresponding C-α (δ_C_ 122.8) and C-β (136.1) were identified from the HSQC spectrum (Figure S5, Supplementary Materials). The presence of two methoxy and a 2,2-dimethylpyrano substituents were evident from the NMR spectra (Table 1). Of the two methoxy functionalities observed, the ^13^C-NMR signal of one was deshielded (δ_C_ 62.9) suggesting *diortho*-substitution. This methoxy group (δ_H_ 3.77) showed a NOE correlation to H-β (δ_H_ 8.15) and H-4′′ (δ_H_ 6.55), and was accordingly placed at C-2′ (Figure S3, Supplementary Materials). The second methoxy group (δ_H_ 3.88, δ_C_ 55.9) showed a NOE correlation with the aromatic singlet δ_H_ 6.25 (H-5′), and hence was placed at C-6′, supported by the HMBC correlations (Figure S6, Supplementary Materials) of H-5′ (δ_H_ 6.25) with C-1′ (δ_C_ 110.5), C-2′ (δ_C_ 161.2), C-3′ (δ_C_ 108.2), and C-4′ (δ_C_ 157.0). The HMBC correlations of H-α (δ_H_ 7.96) with C-1′ (δ_C_ 110.5), C=O (δ_C_ 192.0) and those of H-β (δ_H_ 8.15) with C-6′ (δ_C_ 157.7), C-2′ (δ_C_ 161.2), C-α (δ_C_ 122.8) and C=O (δ_C_ 192.0) suggested that compound **1** is a retrochalcone [14,15,16]. The high chemical shift of H-2/6 of ring A (δ_H_ 8.01), which showed a HMBC correlation with the carbonyl carbon (δ_C_ 192.0), and the lack of NOE between H-2/6 (δ_H_ 8.01) and H-β (δ_H_ 8.15) suggested that the carbonyl is adjacent to ring A [17]. This ring is unsubstituted, as indicated by the COSY correlations connecting the H-2/6 (δ_H_ 8.01), H-3/5 (δ_H_ 7.47) and H-6 (δ_H_ 7.53) spin system. The connection of the 2,2-dimethylpyrano group (C ring) to the B ring via the bridging C-3′ and C-4′ atoms was revealed by the HMBC correlations of H-4′′ (δ_H_ 6.55) with C-3′ (δ_C_ 108.2) and C-4′ (δ_C_ 157.0), and by that of H-3′′ (δ_H_ 5.55) with C-3′ (δ_C_ 108.2). It was further confirmed by the NOE of H-4′′ (δ_H_ 6.55) and MeO-2′ (δ_H_ 3.77). The HMBC correlations of H-3′′ (δ_H_ 5.55) with Me-2′′ (δ_C_ 28.1) and C-2′′ (δ_C_ 77.0) along with the NOE of H-3′′ (δ_H_ 5.55) with Me-2′′ (δ_H_ 1.44) defined the constitution of the C ring. Thus, on the basis of its spectroscopic data, compound **1** was characterized as *E*-2′,6′-dimethoxy-3′,4′-(2′′,2′′-dimethyl)pyranoretrochalcone, and was assigned the trivial name aequichalcone A.

Compound **2** was isolated as a colorless paste, and was assigned the molecular formula C_22_H_22_O_4_ based on HRESIMS ([M + H]^+^ *m*/*z* obs 351.1585, calcd 350.1590) and NMR (Table 1) analyses. Similar to compound **1**, the NMR signals δ_H_ 6.94 (d, *J* = 12.6 Hz) and δ_H_ 6.57 (d, *J* = 12.6 Hz), corresponding to H-α and H-β, respectively, suggested a chalconoid skeleton, in this case, however, with a *Z*-double bond configuration. Ring B of **2** was observed to be comparable to that of **1**, with two methoxy groups at C-2′ (δ_H_ 3.47, δ_C_ 54.9) and C-6′ (δ_H_ 3.67, δ_C_ 61.8), and a 2,2-dimethylchromene ring C connected to ring B via the bridging C-3′ (δ_C_ 107.7) and C-4′ (δ_C_ 155.2) atoms. The substitution pattern of ring C was confirmed by HMBC and NOESY correlations (Figures S14–S12, Supplementary Materials), as described above for **1**. Ring A of **2** was unsubstituted, and thus the only difference between **1** and **2** was the geometry of their α,β-double bond, reflected by the ^3^*J*_HαHβ_ = 16.0 Hz vs. 12.6 Hz, and the strong NOE of H-α and H-β observed for **2** (Figure S12, Supplementary Materials) but not for **1** (Figure S3, Supplementary Materials). Therefore, compound **2** was characterized as *Z*-2′,6′-dimethoxy-3′,4′-(2′′,2′′-dimethyl)pyranoretrochalcone, and was given the trivial name aequichalcone B.

Despite being geometrical isomers at one double bond, the chemical shifts of **1** and **2** were substantially different. Particularly, H-α (δ_H_ 7.96) and H-β (δ_H_ 8.15) of the *E*-isomer **1** were deshielded compared to those of the *Z*-isomer (H-α δ_H_ 6.94; H-β δ_H_ 6.57). Moreover, the carbonyl of **2** was deshielded (δ_C_ 194.4) compared to that of compound **1** (δ_C_ 192.0). These data suggested that due to steric crowding, the α,β-unsaturated carbonyl system of **2** was distorted and did not possess coplanar aromatic rings, decreasing the extent of the conjugation. The shielding of OMe-2′ (δ_H_ 3.47) and OMe-6′ (δ_H_ 3.67) of **2** further indicates that ring B was most likely perpendicular to the α,β-unsaturated system, and accordingly the methoxy groups experience the anisotropy effect of the α,β-unsaturated carbonyl system. Compound **2** was colorless and showed only a benzenoid absorption band at λ_max_ 245 nm, while compound **1** was yellow and possessed the characteristic UV spectrum of chalconoids with λ_max_ at 240, 290 and 370 nm, further corroborating the above hypothesis. Such distortion was reported earlier for *Z*-preacansone A [9] and for methyltepanone [18].

Upon standing at room temperature in acetone-*d*_6_ solution for days, compound **1** was observed by ^1^H-NMR to slowly convert to compound **2** (1:2.5 mixture of **1** and **2**, following 48 h). Diabatic photoisomerization processes are known to yield a photostationary state containing a mixture of *Z* and *E* isomers [19,20]. Although rarely discussed, for numerous olefins the *Z* isomer has been reported to be stabilized by hydrophobic forces over the corresponding *E* isomer [21,22]. Photoisomerization of *E*-enonones, yielding a mixture of *Z* and *E* isomers, similar to our observation, has been previously reported [23]. Consequently, we cannot rule out that **2** may have been enriched, or formed, due to a light-induced isomerization during the extraction and separation process. A similar phenomenon has been observed for the retrochalconoids preacansone A and methyltepanone isolated from *Tephrosia pumila* [9] and *Ellipeia cuneijblia* [18], respectively.

Compound **3** was isolated as a yellow paste, and assigned the molecular formula C_24_H_28_O_7_ based on HRESIMS ([M + H]^+^ obs *m*/*z* 429.1905, calcd 429.1908) and NMR analyses (Table 2). It showed UV absorption at λ_max_ 225 and 334 nm, which along with its NMR data suggested it to be a chalconoid derivative as well. The high similarity of its NMR spectra with those of praecansone B (**7**) [8] suggested **3** to be a β-hydroxychalcone. Its H-α, olefinic proton (δ_H_ 6.57) showed a HMBC correlation with C-1 (δ_C_ 135.0), C-1′ (δ_C_ 114.6), C-9 (δ_C_ 188.1). Based on the arguments described for **1** above, ring A of **3** was assumed to be unsubstituted. Its ring B was substituted with two methoxy groups at C-2′ (δ_H_ 3.87, δ_C_ 62.6) and C-6′ (δ_H_ 3.82, δ_C_ 55.9), as revealed by the HMBC correlations of H-5′ (δ_H_ 6.27) of this ring with C-1′ (114.6), C-3′ (107.2), C-4′ (155.8), C-6′ (158.7) and the NOE observed between H-5′ (δ_H_ 6.27) and MeO-6′ (δ_H_ 3.82) (Figure S20, Supplementary Materials). In contrast to the structurally closely related compound **7** which possesses a 2,2-dimethylchromene ring C, that of **3** is saturated and substituted. Thus, protons H-3′′ and H-4′′ of **3** are not olefinic, but showed ^1^H-NMR signals at δ_H_ 3.86 and δ_H_ 4.40, respectively. The chemical shift of these along with that of the corresponding carbon signals at δ_C_ 70.3 (C-3′′) and δ_C_ 72.8 (C-4′′) suggested that both are oxygenated. Whereas C-3′′ (δ_C_ 70.3) was substituted with a hydroxy group, C-4′′ (δ_C_ 72.8) bears an ethoxy functionality (δ_H_ 3.75, 2H, q; δ_C_ 64.9; δ_H_ 1.24, 3H, t; δ_C_ 15.3). The placement of the ethoxy group at C-4′′ was based on the HMBC correlation of its oxymethylene protons (δ_H_ 3.75) with C-4′′ (δ_C_ 155.8) and that of H-4′′ (δ_H_ 4.40) with C-2′ (δ_C_ 160.2). The *gauche* coupling (*J* = 2.8 Hz) of H-3′′ (δ_H_ 3.86) and H-4′′ (δ_H_ 4.40) revealed their *cis* configuration. Ethoxy substitution is unusual among natural products, yet **3** is not the first to possess a 4′′-ethoxy-3′′-hydroxydihydropyran ring [24]. On the basis of the above spectroscopic data, and by comparison with that of praecansone B (**7**), compound **3** was characterized as 3′′,4′′-*cis-*4′′-ethoxy-3′′-hydroxypraecansone B and given the trivial name aequichalcone C.

Compound **4** was isolated as an amorphous solid, and assigned the molecular formula C_17_H_10_O_6_ based on HRESIMS ([M + H]^+^ *m*/*z* obs 310.0512, calcd 310.0472) and NMR (Table 3) analyses. 

It showed characteristic UV (λ_max_ 225, 337 and 353 nm), ^1^H-NMR (δ_H_ 5.54, s, CH_2_-6) and ^13^C-NMR (δ_C_ 65.8, CH_2_-6; δ_C_ 119.0, C-6a; δ_C_ 147.0, C-11a) features for a pterocarpene skeleton [25,26]. The NMR spectra indicated the presence of two methylenedioxide groups (δ_H_ 5.97, δ_C_ 101.8 and δ_H_ 6.00, δ_C_ 101.7), connected at the bridging C-3 and C-4, and C-8 and C-9 of the pterocarpene skeleton, as revealed by the HMBC correlations of 3,4-OCH_2_O- (δ_H_ 6.00) to C-3 (δ_C_ 149.5) and C-4 (δ_C_ 134.5) and 8,9-OCH_2_O- (δ_H_ 5.97) to C-8 (δ_C_ 144.1) and C-9 (δ_C_ 146.1). Moreover, the two *ortho*-coupled (*J* = 8.0 Hz) aromatic protons at δ_H_ 6.98 and δ_H_ 6.50, and the two *para*-oriented aromatic protons at δ_H_ 7.02 and δ_H_ 6.76 indicated that rings A and D were disubstituted. The substitution pattern of ring A was determined based on the HMBC correlation of H-1 (δ_H_ 6.98) with C-11a (δ_C_ 147.0) and the oxygenated C-3 (δ_C_ 149.5) along with the *ortho*-coupling of H-1 (δ_H_ 6.98) and H-2 (δ_H_ 6.50), which is consistent with the HMBC-based placement (*vide supra*) of the methylenedioxide group at C-3 (δ_C_ 149.5) and C-4 (δ_C_ 134.5). The *para*-orientation of the aromatic protons H-7 (δ_H_ 7.02) and H-10 (δ_H_ 6.76) of ring D is consistent with the second methylenedioxide group being placed at C-8 (δ_C_ 144.9) and C-9 (δ_C_ 146.1). Assignation of the carbons of rings B and C was based on the HMBC correlations of H-1 (δ_H_ 6.98), H-6 (δ_H_ 5.54), H-7 (δ_H_ 7.02) and H-10 (δ_H_ 6.76) (Table 3). On the basis of the above spectroscopic evidence, this new compound (**4**) was characterized as 3,4:8,9-dimethyl-enedioxypterocarpene.

The crude CH_2_Cl_2_/CH_3_OH (1:1) extract of the roots of *Tephrosia aequilata* resulted in 100% growth inhibition of the chloroquine-sensitive (3D7) strain of *Plasmodium falciparum* at 10 µg/mL. The compounds isolated from this extract were also tested for antiplasmodial activity using a previously established protocol [27,28]. Compound **3** showed good (IC_50_ < 5 µM), while all other compounds showed moderate (IC_50_ 6–9 µM) [29] antiplasmodial activities (Table 4). These activities are in the same range of those reported for licochalcone A (IC_50_ 4.17 µM [29] against the 3D7 strain), a retrochalcone which is also known for its in vivo antimalarial activity and for enhancing the activity of artemisinin in vitro [29]. It is therefore of value to investigate the chalconoids of this plant for similar activities. None of the compounds showed cytotoxicity against the HEK-293 human embryonic kidney cell line, up to a concentration of 40 µM, showing that the observed antiplasmodial activities are not due to general toxicity.

## 3. Materials and Methods

### 3.1. General Experimental Procedures

UV spectra were recorded on a Specord S600 (Analytik Jena AG, Jena, Germany) spectrophotometer, optical rotations were measured on PerkinElmer 341-LC (PerkinElmer, Wellesley, MA, USA) whereas CD experiments were run on a Jasco J-715 spectropolarimeter (Jasco, Corp., Tokyo, Japan). NMR spectra were acquired on Bruker Advance 600 or a Bruker Advance III HD 800 spectrometer (Bruker BioSpin AG, Fällanden, Switzerland), using the residual solvent signal as reference. EI-MS spectra were obtained on a Micromass GC-TOF mass spectrometer (Micromass, Wythenshawe, Waters Inc., Manchester, UK), using direct inlet, and 70 eV ionization voltage. TLC was carried out on Merck pre-coated Silica gel 60 F254 plates (Merck, Darmstadt, Germany). Column chromatography was run on silica gel 60 (70–230 mesh). Gel filtration was done on Sephadex LH-20 (Fluka, Buchs, Switzerland). Preparative HPLC was carried out on a Waters 600E instrument (Waters Corp, Milford, MA, USA) using the Chromulan (Pikron Ltd., Praha, Czech Republic) software and an RP C8 Kromasil^®^ (250 mm × 55 mm, Kromasil, Bohus, Sweden) column with a CH_3_OH/H_2_O solvent system. HRESIMS were obtained with a Q-TOF-LC/MS spectrometer (Stenhagen Analyslab AB, Gothenburg, Sweden) using a 2.1 mm × 30 mm, 1.7 μm RPC18 column and a H_2_O:CH_3_CN gradient system (5:95−95:5 gradient and 0.2% formic acid).

### 3.2. Plant Material

The roots of *Tephrosia aequilata* were collected in May, 2013 from the Kilungu hills in Makueni County, Kenya. The plant specimen was identified by Mr. Patrick C. Mutiso of the University Herbarium, School Biological Sciences, University of Nairobi where voucher specimen (Mutiso-841/May 2013) has been deposited.

### 3.3. Extraction and Isolation

The air dried and ground roots of *Tephrosia aequilata* (2 kg) were extracted with CH_2_Cl_2_/MeOH, 1:1 (5 × 1.5 L) by percolation. The extract was filtered and the solvent removed under vacuum using a rotary evaporator at 50 °C to yield 120 g dark brown paste. The extract was diluted with methanol and extracted with *n*-hexane to remove the fat. The methanol layer (80 g) was subjected to column chromatography on Silica gel (600 g) eluting with *n*-hexane containing increasing percentages of EtOAc. The fraction eluted with 1% EtOAc in *n*-hexane was washed with acetone to yield 3,4:8,9-dimethylenedioxypterocarpene (**4**, 100 mg) as colorless solid. The acetone soluble portion was subjected to column chromatography on Sephadex LH-20 (CH_2_Cl_2_/CH_3_OH, 1:1) to yield obovatin methyl ether (**5**, 5 mg) [7]. The fraction eluted with 3% EtOAc in *n*-hexane was further subjected to column chromatography on a silica gel (120 g) to yield obovatachalcone (**6**, 20 mg), praecansone B (**7**, 900 mg) and *Z*-praecansone A (**8**, 100 mg) [1,7,30,31]. The fractions eluted with 5%–7% EtOAc in *n*-hexane were combined and purified on preparative HPLC (CH_3_OH/H_2_O, gradient elution) to give aequichalcone B (**2**, 20 mg) and aequichalcone A (**1**, 25 mg). The fraction eluted with 7% EtOAc was purified over Sephadex LH-20 (CH_2_Cl_2_/CH_3_OH, 1:1) and was further purified by PTLC (5% EtOAc in *n*-hexane) to give aequichalcone C (**3**, 15 mg). The fraction eluted with 10% EtOAc was purified by PTLC (7% EtOAc in *n*-hexane) to give candidone (**9**, 10 mg) [2]. The fractions eluted with 15%–20% EtOAc in *n*-hexane were combined and subjected to column chromatography over Sephadex LH-20 (CH_2_Cl_2_/CH_3_OH, 1:1) to give isopongaflavone (**10**, 1.2 g) [10,11]. The fraction eluted with EtOAc:MeOH (1:1) was crystallized from MeOH to yield β-sitosterol-3-*O*-glucoside (**11**, 50 mg) [29].

The negative optical rotation of compounds **5** and **8**, [α]_D_ −16.35 (c 0.001, CH_2_Cl_2_) and −21.5 (c 0.001, CH_2_Cl_2_), respectively, is in good agreement with that previously published for the *S*-configuration of these compounds [6].

*E-2′,6′-Dimethoxy-3′,4′-(2*′′*,2*′′*-dimethyl)pyranoretrochalcone* (**1**): Yellow paste. UV (CH_2_Cl_2_) λ_max_: 240, 290 and 370 nm. ^1^H- and ^13^C-NMR (Table 1). ESIMS *m*/*z* 351.7 [M + H]^+^. HRMS [M]^+^ *m*/*z* 350.1506 C_22_H_22_O_4_ (Calculated: 350.1518).

*Z-2′,6′-Dimethoxy-3′,4′-(2*′′*,2*′′*-dimethyl)pyranoretrochalcone* (**2**): Colorless paste. UV (CH_2_Cl_2_) λ_max_: 245 nm. ^1^H- and ^13^C-NMR (Table 1). EIMS *m*/*z* (rel. int.) 397 [M]^+^ (100), 325 (23), 383 (20), 297 (15). HRMS [M]^+^ *m*/*z* 351.1586 C_22_H_22_O_4_ (Calculated: 351.1596). 

*3*′′*,4*′′*-cis-4*′′*-Ethoxy-3*′′*-hydroxypraecansone B* (**3**): Yellowish oil. UV (CH_2_Cl_2_) λ_max_: 225, 334 nm. CD (CH_2_Cl_2_) λ nm (Δε; M^−^^1^ cm^−^^1^): (−3.7)_403_; (0.9)_297_; (2.4)_209_. [α]_D_ −18.87° (*c* 0.001, CH_2_Cl_2_). ^1^H- and ^13^C-NMR (Table 2) EIMS *m*/*z* (rel. int.) 397 [M]^+^ (100), 325 (23), 383 (20), 297 (15). HRMS [M]^+^ *m*/*z* 429.1905 C_24_H_28_O_7_ (Calculated: 429.1913). 

*3,4:8,9-Dimethylenedioxypterocarpene* (**4**): Colorless crystal. M.p. 198–200 °C; UV (CH_2_Cl_2_) λ_max_: 225, 337, 353 nm. ^1^H- and ^13^C-NMR (Table 3) EIMS *m*/*z* (rel. int.) 397 [M]^+^ (100), 325 (23), 383 (20), 297 (15). HRMS [M]^+^ *m*/*z* 310.0512 C_17_H_10_O_6_ (calculated: 310.0477).

### 3.4. Plasmodium Falciparum Culture

In vitro parasite culture of the *P. falciparum* (strain 3D7) was maintained in RPMI with 10 mM Hepes (Life Technologies, Nærum, Denmark), 50 μg/mL hypoxanthine (Sigma, Saint Louis, MO, USA) and 5% human serum from male AB plasma and 2.5 mg/mL AlbuMAX II^®^ (Life Technologies, Paisley, UK). Human 0+ erythrocytes were provided bythe Australian Red Cross Blood Bank (Agreement No: 13-04QLD-09). The parasites were maintained at 2%–8% parasitaemia (% P) at 5% haematocrit (% H), and incubated at 37 °C, 5% CO_2_, 5% O_2_, 90% N_2_ and 95% humidity.

### 3.5. Plasmodium falciparum Growth Inhibition Assay

A well-established asexual *P. falciparum* imaging assay was used to determine parasite growth inhibition according to the procedure described by Duffy and Avery [28]. Briefly, 2% or 3% parasite (3D7) and 0.3% hematocrit in a total assay volume of 50 μL were incubated in the presence of compounds for 72 h at 37 °C and 5% CO_2_, in poly-d-lysine-coated Cell Carrier Imaging plates. After incubation, plates were stained with DAPI (6,4′-diamidino-2-phenylindole) in the presence of saponin and Triton X-100, and incubated in the dark for a further 5 h at room temperature before imaging on the OPERA HTS confocal imaging system (PerkinElmer, Waltham, MA, USA). The digital images obtained were analyzed using the PerkinElmer Acapella spot detection software (version 2.0, PerkinElmer). We counted the spots in fulfilling the criteria established for a stained parasite. The % inhibition of parasite replication was calculated, using DMSO and artemisinin as control data.

Human red blood cells for plasmodium culture were provided by the Australian Red Cross Blood Bank in accordance with their routine MTA for nonclinical blood product supply. All work undertaken is covered by the approval from the Griffith University Biosafety and Human Ethics Committee, GU ref no. ESK/03/12/HREC.

### 3.6. Cytotoxicity Assays

The cytotoxicity of compounds against HEK-293 cells was assessed in dose response using a resazurin-based viability assay. HEK-293 cells were grown in DMEM medium (Life Technologies), containing 10% fetal calf serum (FCS; Gibco), trypsinised, counted and seeded at 2000 cells per well in 45 μL media into TC-treated 384-well plates (Greiner) and left to adhere overnight at 37 °C, 5% CO_2_ and 95% humidity. Test compounds were prepared by diluting 1 in 25 in sterile water and then another 1 in 10 dilution, to give a top final test concentration of 40 μM, 0.4% DMSO. Plates were incubated for 72 h at 37 °C, 5% CO_2_ and 95% humidity, the media was removed and replaced by 35 μL of 44 μM resazurin in DMEM without FCS. The plates were incubated for another 4–6 h at 37 °C, 5% CO_2_ and 95% humidity, before reading on an EnVision^®^ Plate Reader (PerkinElmer) using fluorescence excitation/emission settings of 530 nm/595 nm. The % growth was standardized to controls (40 μM puromycin as positive and 0.4% DMSO as negative control) using the software Microsoft^®^ Excel 2013. Statistical analysis, including IC_50_ determination and graphical output, was done in GraphPad Prism^®^ 6 (GraphPad Software, San Diego, CA, USA) using nonlinear regression variable slope curve fitting. The experiments were carried out in two independent biological replicates, each consisting of two technical replicates.

## 4. Conclusions

Four new flavonoids along with seven known natural products were identified from the CH_2_Cl_2_/CH_3_OH (1:1) root extract of *T. aequilata*. Most of these compounds showed good to moderate antiplasmodial activities against the chloroquine-sensitive (3D7) strain of *Plasmodium falciparum*.

## Figures and Tables

**Table 1:** ^1^H (800 MHz) and ^13^C (200 MHz) NMR data for aequichalcone A (**1**) and B (**2**) acquired in CDCl_3_ at 25 °C.

Position	1				2			
	δ_C_	δ_H_, m, (*J* in Hz)	HMBC	NOE	δ_C_	δ_H_, m, (*J* in Hz)	HMBC	NOE
1	139.0	-			137.6	-		
2/6	128.7	8.01 dd (7.7, 1.4)	C-3/5, C-4, C-7		128.7	7. 86 dd (6.9,1.4)	C-3/5, C-4, C-7	H-α, H-3/5
3/5	128.0	7.47 dd (7.7, 7.7)	C-1, C-2/6		128.0	7.34 dd (7.4,6.9)	C-1, C-2/6,	
4	132.2	7.53 tt (7.7, 1.4)	C-2/6, C-3/5		132.1	7.43 tt (7.4,1.4)	C-2/6, C-3/5	
7	192.0				194.4			
α	122.8	7.96 d (16.0)	C-7, C-1′		127.3	6.57 d (12.6)	C-1′, C-7	H*-*β
β	136.1	8.15 d (16.0)	C-α, C-7, C-6′, C-2′	OMe-6′	130.0	6.94 d (12.6)	C-1′, C-2′, C-6′, C-7	H-α
1′	110.5	-			111.4	-		
2′	161.2	-			155.0	-		
3′	108.2	-			107.7	-		
4′	157.0	-			155.2	-		
5′	96.4	6.25 *s*	C-1′, C-2′, C-3′, C-4′	OMe-6′	96.0	6.01 *s*	C-1′, C-3′, C-4′, C-6′	OMe-6′
6′	157.7	-			157.6	-		
2′′	77.0	-			76.6	-		
3′′	128.4	5.55 d (9.9)	C-2′′, C-3′, 2′′-Me_2_	2′′-Me_2_	127.3	5.44 d (10.0)	C-2′′, C-3′, 2′′-Me_2_	H-4′′
4′′	116.5	6.55 d (9.9)	C-2′, C-3′, C-4′, C-2′′	OMe-2′	116.8	6.41 d (10.0)	C-′ C-3′, C-4′, C-2′′	H-3′′, OMe-6′
2′′-Me_2_	28.1	1.44 s	C-2′′, C-3′′		27.9	1.37 s	C-2′′, C-3′′	
OMe-2′	62.3	3.77 s	C-2′	H-3′	54.9	3.47 s	C-2′	
OMe-6′	55.9	3.88 s	C-6′	H-4′′, H-α, H*-*β	61.8	3.67 s	C-6′	H-α, H*-*β

**Table 2:** ^1^H (600 MHz) and ^13^C (150 MHz) NMR data for aequichalcone C (**3**) acquired in CD_2_Cl_2_ at 25 °C.

Position	δ_C_	δ_H_, m, *J* in Hz	HMBC	NOE
1	135.0	-		
2/6	127.0	7. 97 m	C-2/6, C4, C-7	H-8
3/5	128.6	7.52 m	C-2/6, C-1	
4	132.2	7.59 m	C-1, C-3/5, C-2/6	
7	182.2			
8α	100.6	6.57 *s*	C-1, C-1′, C-7, C-9,	
9β	188.1			
1′	114.6			
2′	160.2			
3′	107.2			
4′	155.8			
5′	95.9	6.27 *s*	C-1′,C-3′, C-4′, C-6′, C-9, C-4′′	OMe-6′
6′	158.7			
2′′	77.5			
3′′	70.3	3.86 d (2.8)	C-4′′	2′′-Me_2_
4′′	72.8	4.40 d (2.8)	C-2′, C-3′, C-4′, C-2′′, C-3′′, C-2′′′	
O*CH*_2_CH_3_	64.8	3.75 m	OCH_2_*CH*_3_, C-4′′	
OCH_2_*CH*_3_	15.3	1.25 t (7.0, 14.0)	O*CH*_2_CH_3_	
2′′-Me_2_	24.8 23.3	1.47 s 1.49 s	C-2′′, C-3′′	
OMe-2′	62.6	3.87 s		
OMe-6′	55.9	3.82 s		
OH-9		16.37		

**Table 3:** ^1^H (600 MHz) and ^13^C (150 MHz) NMR data for 3,4:8,9-dimethylenedioxypterocarpene (**4**) acquired in CD_2_Cl_2_ at 25 °C.

Position	δ_C_	δ_H_, m, (*J* in Hz)	HMBC
1	113.3	6.98 d (8.0)	C-3, C-4a, C-11a
2	101.8	6.50 d (8.0)	C-3, C-4, C-11b,
3	149.5		
4	134.5		
4a	137.0		
6	65.8	5.54 s	C-4a, C-6a, C-6b, C-11a, C-11b (w)
6a	119.0		
6b	107.3		
7	93.8	7.02 s	C-6a, C-8, C-9, C-10a
8	144.9		
9	146.1		
10	97.3	6.76 s	C-6b, C-7 (w), C-8, C-9, C-10a
10a	150.3		
11a	147.0		
11b	112.5		
3,4-OCH_2_O	101.7	6.00 s	C-3, C-4
8,9-OCH_2_O	101.8	5.97 s	C-8, C-9

**Table 4:** In vitro antiplasmodial activities of isolated compounds and against 3D7 strains of *P. falciparum*.

Samples	IC_50_, μM
Aequichalcone A (**1**)	9.20 ± 1.42
Aequichalcone B (**2**)	9.75 ± 0.81
Aequichalcone C (**3**)	2.48 ± 0.22
3,4:8,9-Dimethylenedioxypterocarpene (**4**)	> 40
Obovatachalcone (**6**)	4.23 ± 1.11
Praecansone B (**7**)	4.14 ± 0.26
Praecansone A (**8**)	6.45 ± 0.48
Isopongaflavone (**10**)	8.19 ± 1.48
Chloroquine	0.0047
Artesunate	0.00067

## Supplementary Materials

NMR, MS and UV spectra. Supplementary materials are available free of charge online.
