# Supplementary material for: Three Chalconoids and a Pterocarpene from the Roots of Tephrosia aequilata
Source: Molecules. 2017 Feb 20;22(2):318. doi: 10.3390/molecules22020318 (PMC6155904; doi:10.3390/molecules22020318)
Supplement: Supplementary file 1 [file molecules-22-00318-s001.pdf]

Three Chalconoids and a Pterocarpene from the Roots of Tephrosia aequilata

Yoseph Atilaw, Sandra Duffy, Matthias Heydenreich, Lois Muiva-Mutisya, Vicky M. Avery, Máté Erdélyi, Abiy Yenesew

Table of contents

Figure	Content	Page
**Fig. S1 –S6**	^{1}H; ^{13}C; COSY; NOESY; HSQC & HMBC of compound **1**	S2-S7
**Fig. S7**	HRMS of compound **1**	S8
**Fig. S8**	UV spectrum of compound **1**	S9
**Fig. S9 –S14**	^{1}H; ^{13}C; COSY; NOESY; HSQC & HMBC of compound **2**	S10-S15
**Fig. S15**	HRMS of compound **2**	S16
**Fig. S16**	UV spectrum of compound **2**	S17
**Fig. S17-S22**	^{1}H; ^{13}C; COSY;NOESY; HSQC & HMBC of compound **3**	S18-S23
**Fig. S23**	HRMS of compound **3**	S24
**Fig. S24**	UV spectrum of compound **3**	S25
**Fig. S25-S29**	^{1}H; ^{13}C; COSY; HSQC & HMBC of compound **4**	S26-S30
**Fig. S30**	HRMS of compound **4**	S31
**Fig. S31**	UV spectrum of compound **4**	S32

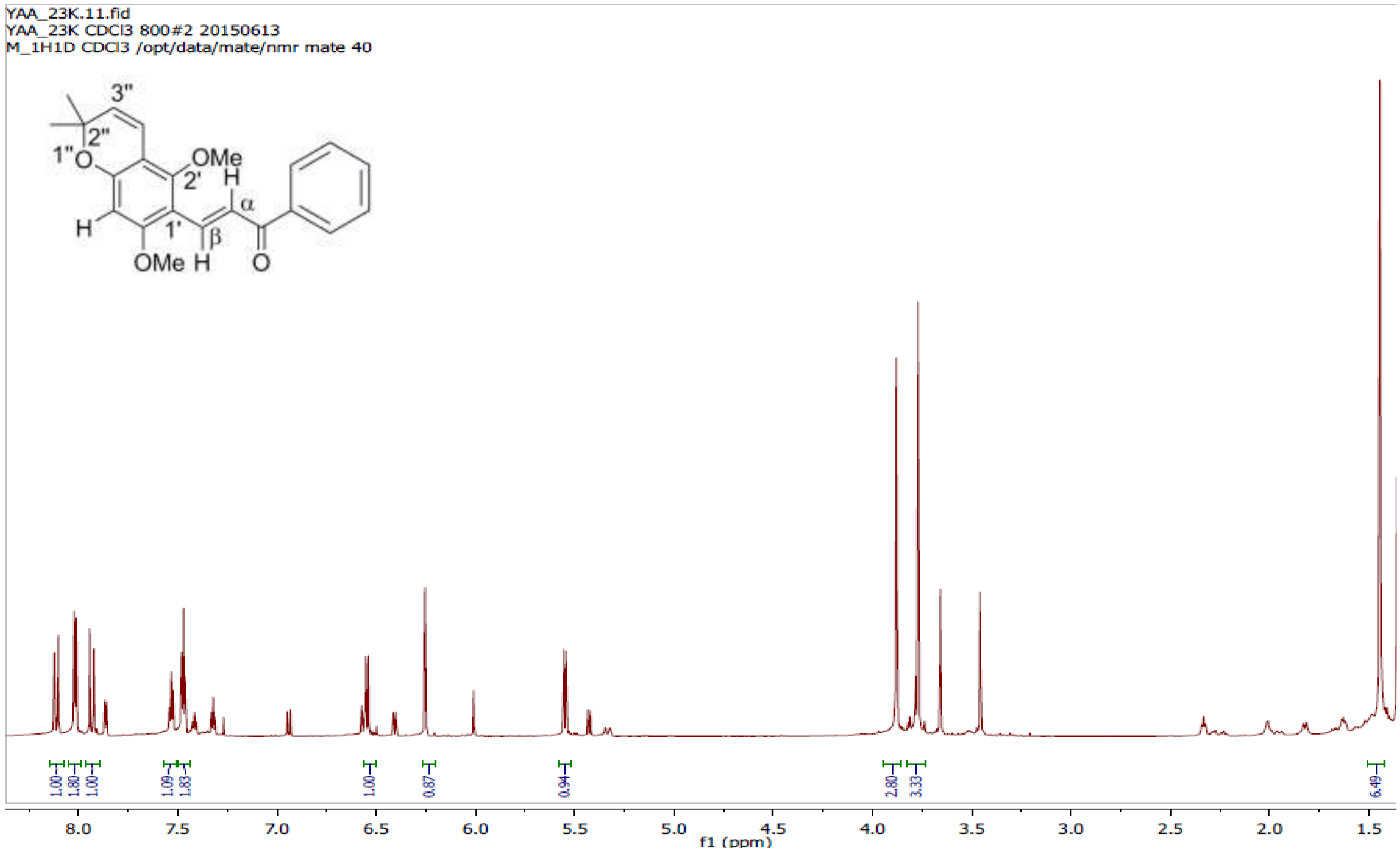

Fig. S1: ^{1}H NMR spectrum of compound **1** (800 MHz; $CDCl_3$)

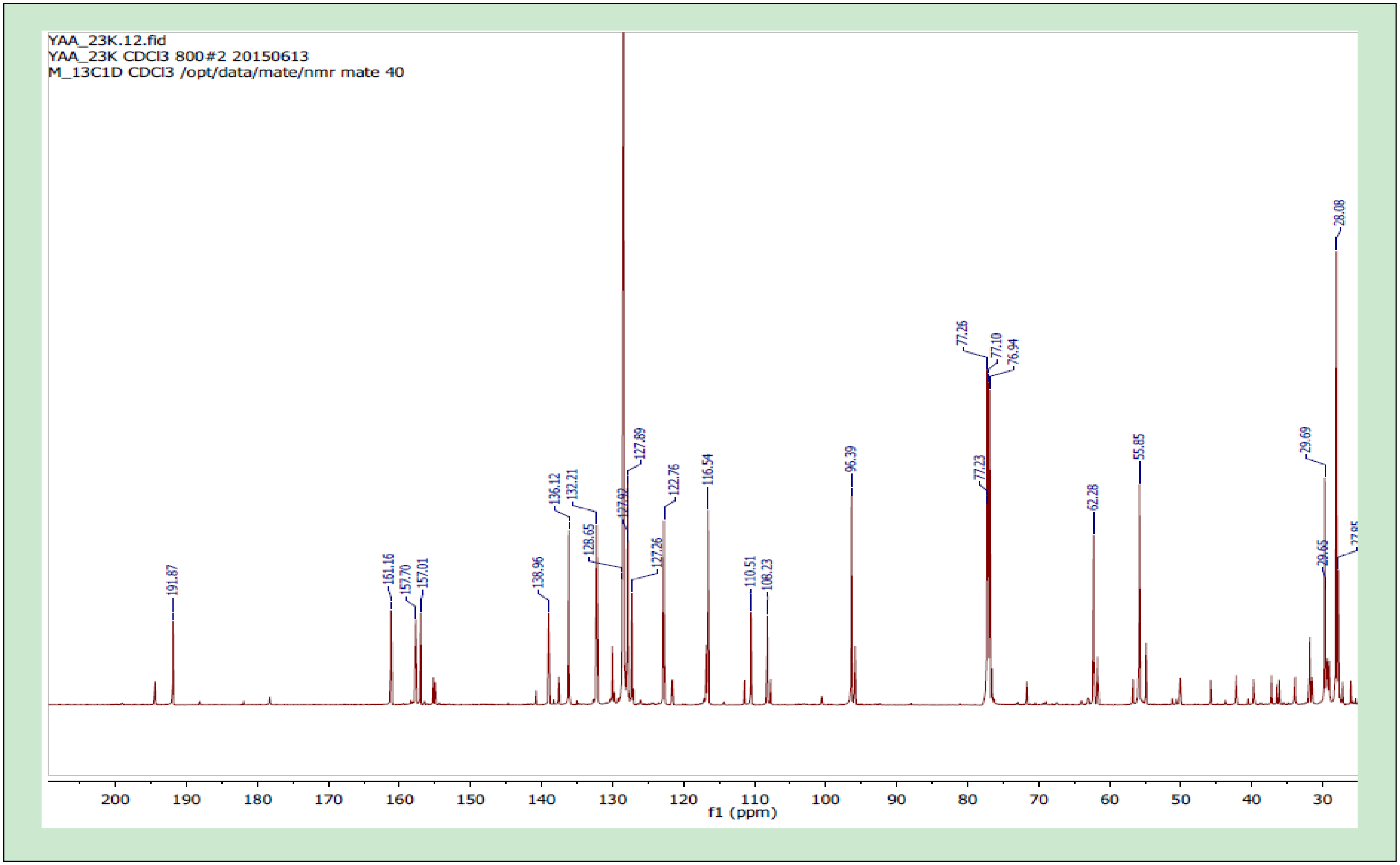

Fig. S2: ^{13}C NMR spectrum of compound **1** (200 MHz; $CDCl_3$)

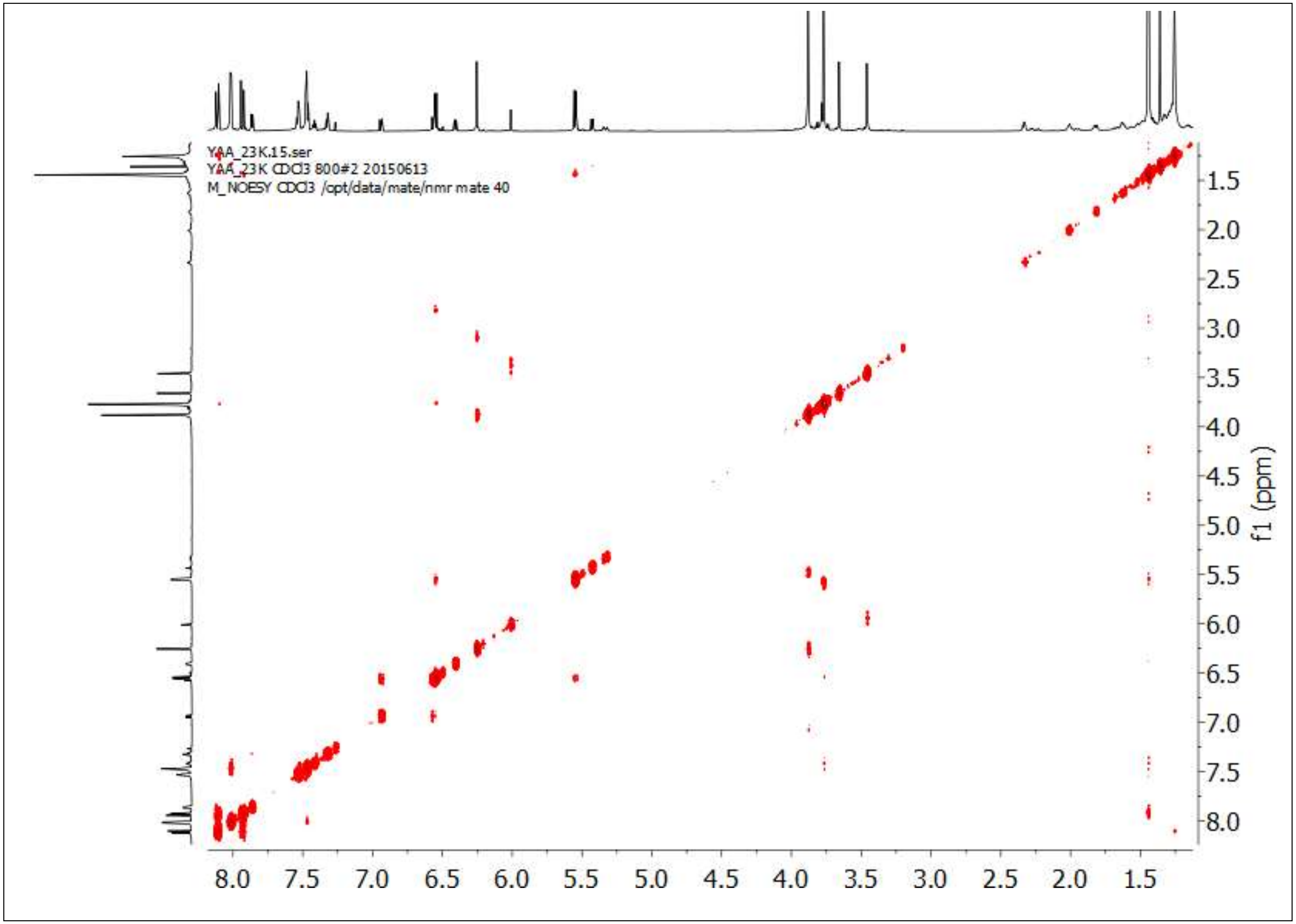

Fig. S3: NOESY spectrum of compound **1** (800 MHz; $CDCl_3$)

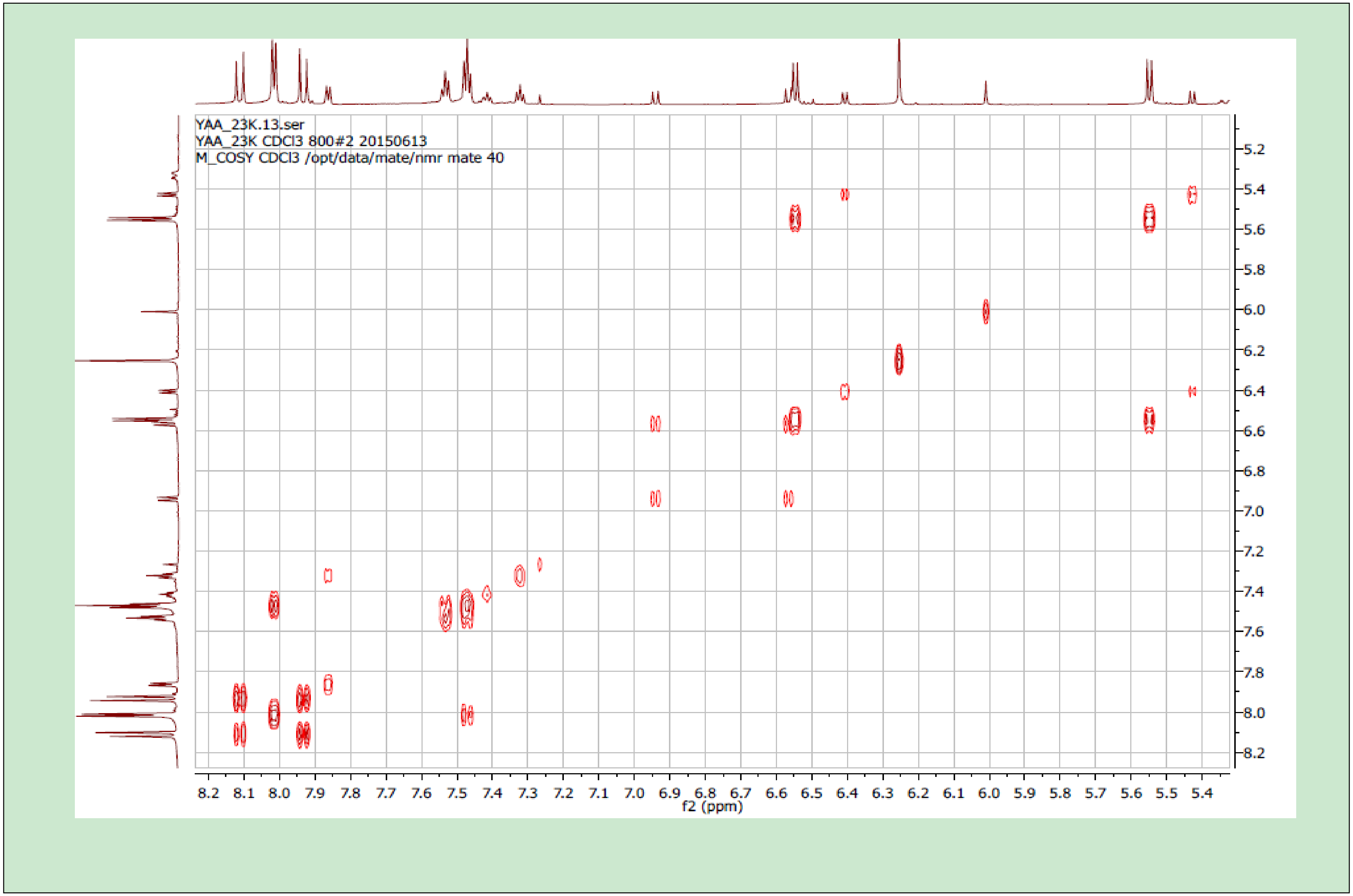

Fig. S4: COSY spectrum of compound **1** (800 MHz; $CDCl_3$)

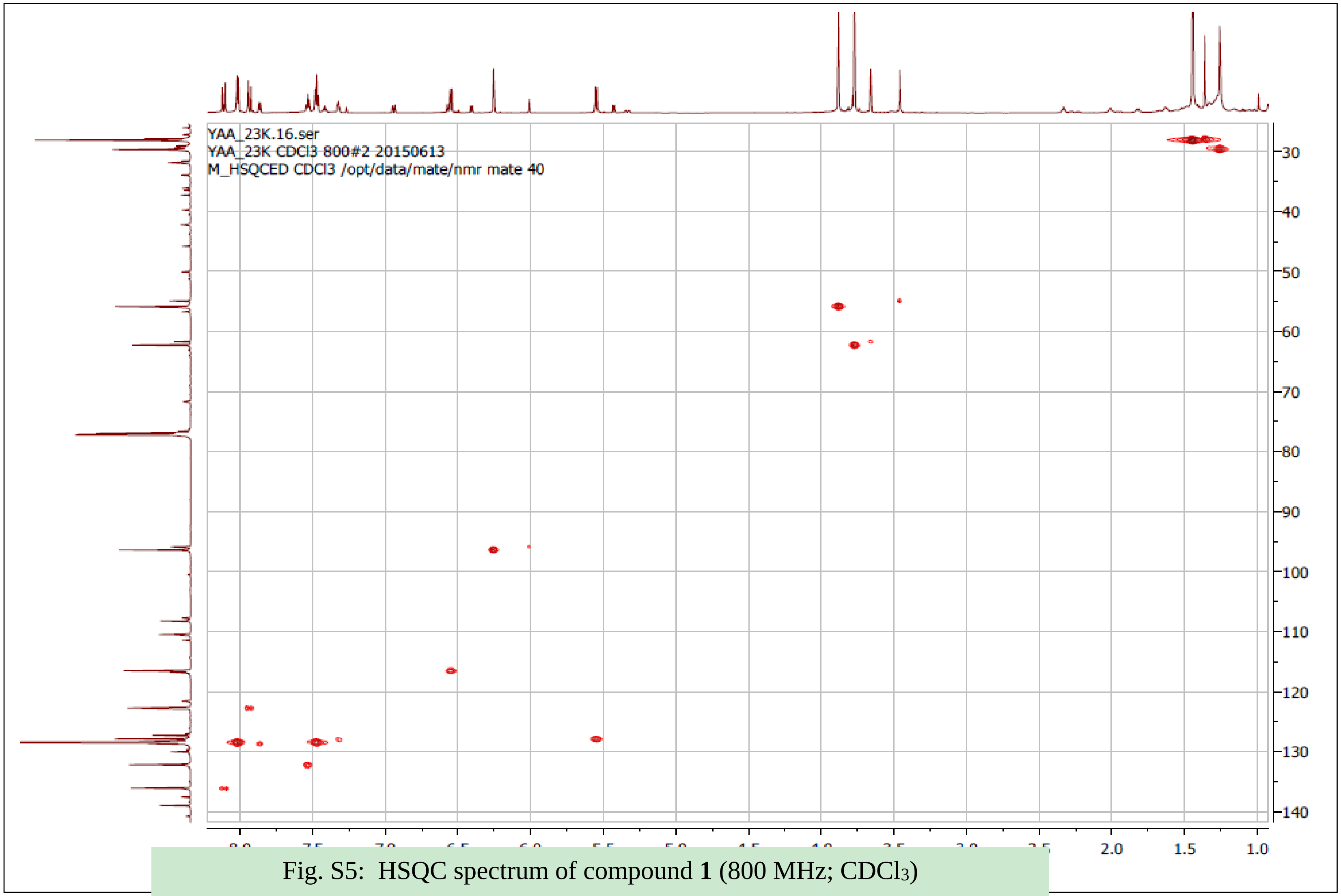

Fig. S5: HSQC spectrum of compound **1** (800 MHz; $CDCl_3$)

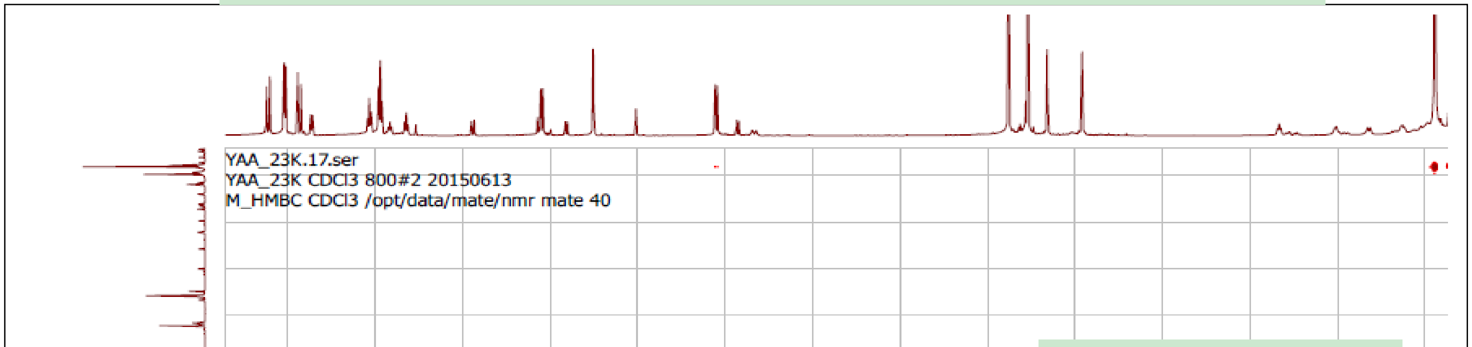

Fig. S6: HMBC spectrum of compound **1** (800 MHz; $CDCl_3$)

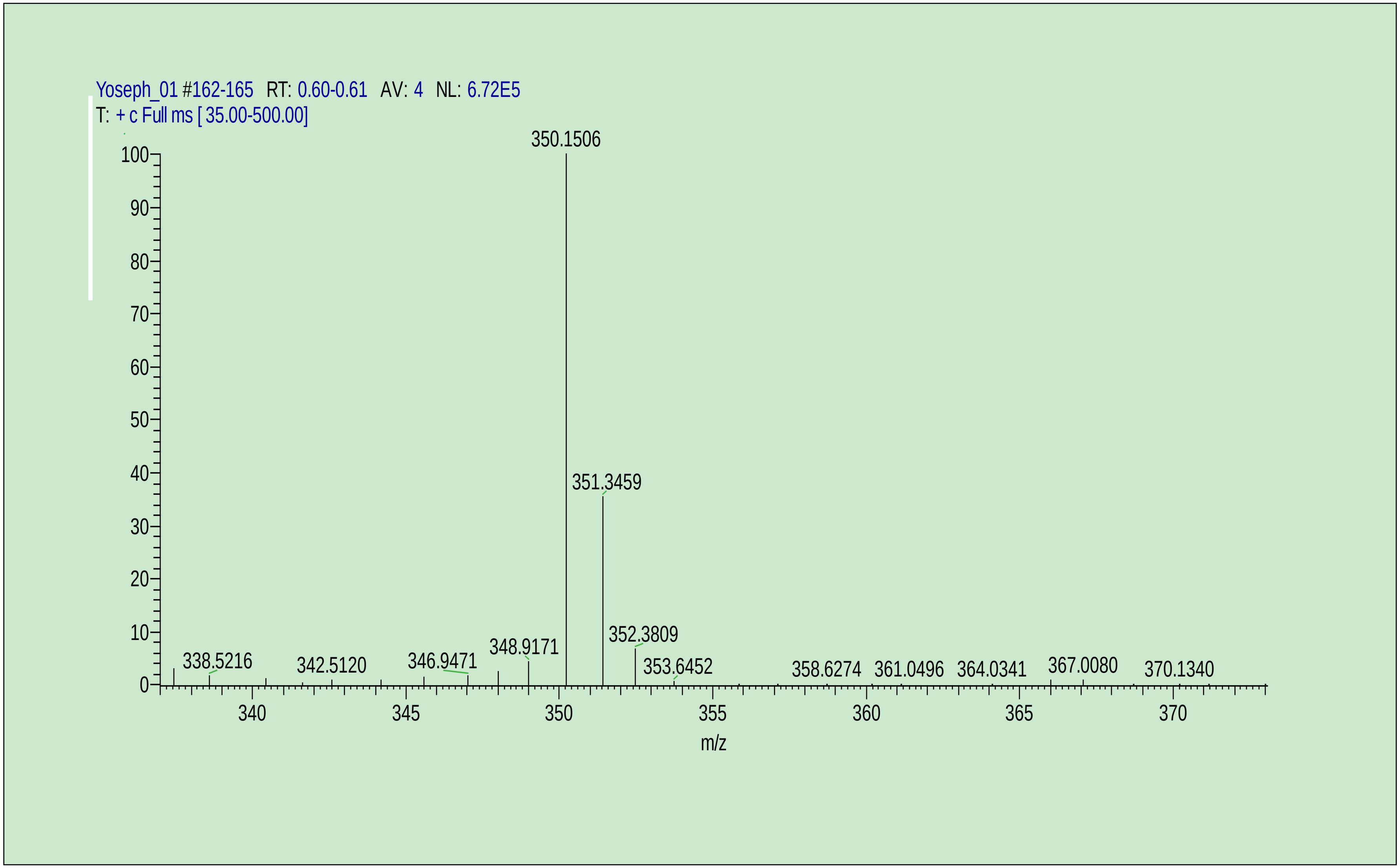

Fig. S7: HRMS of compound **1**

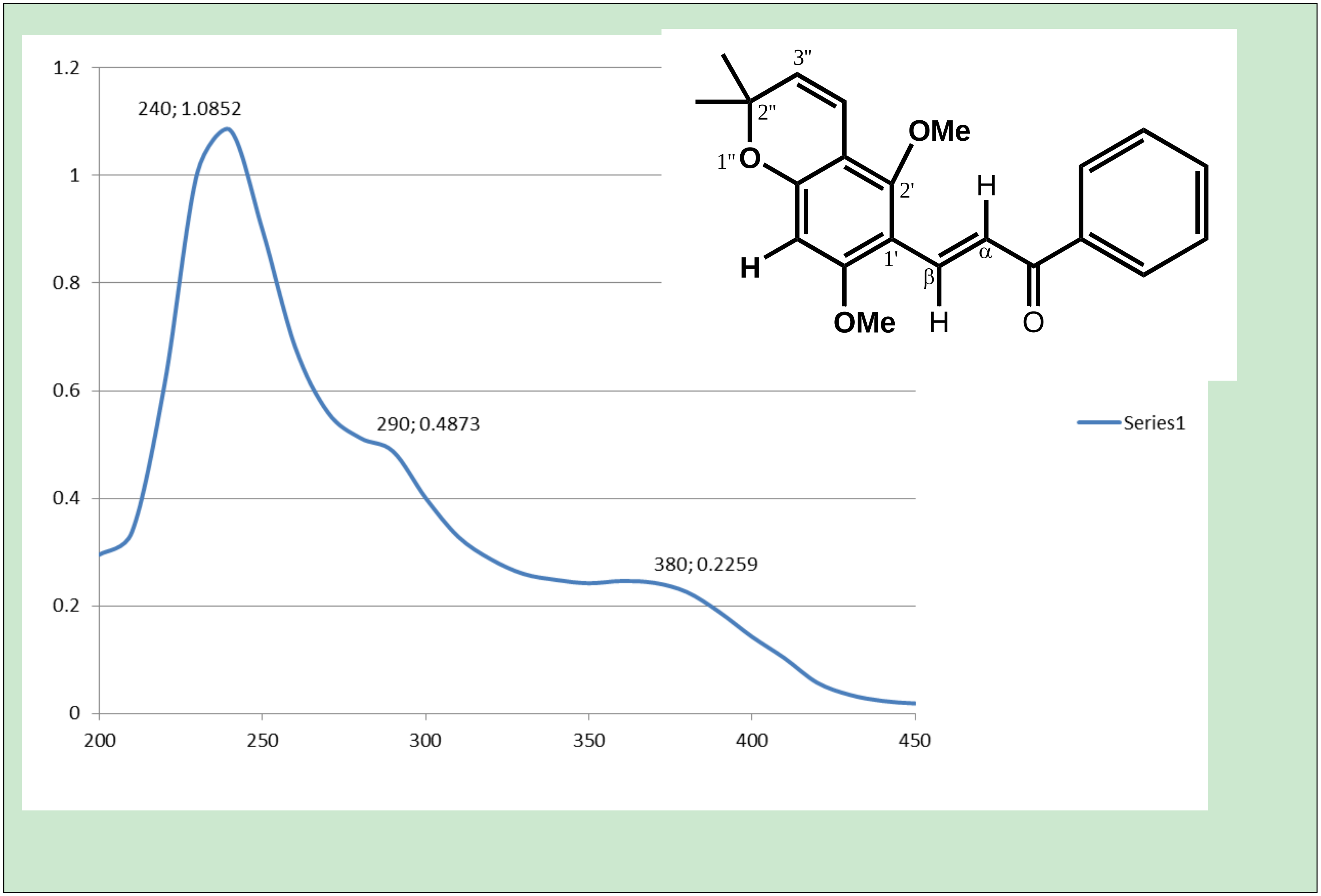

Fig. S8: UV spectrum of compound **1.**

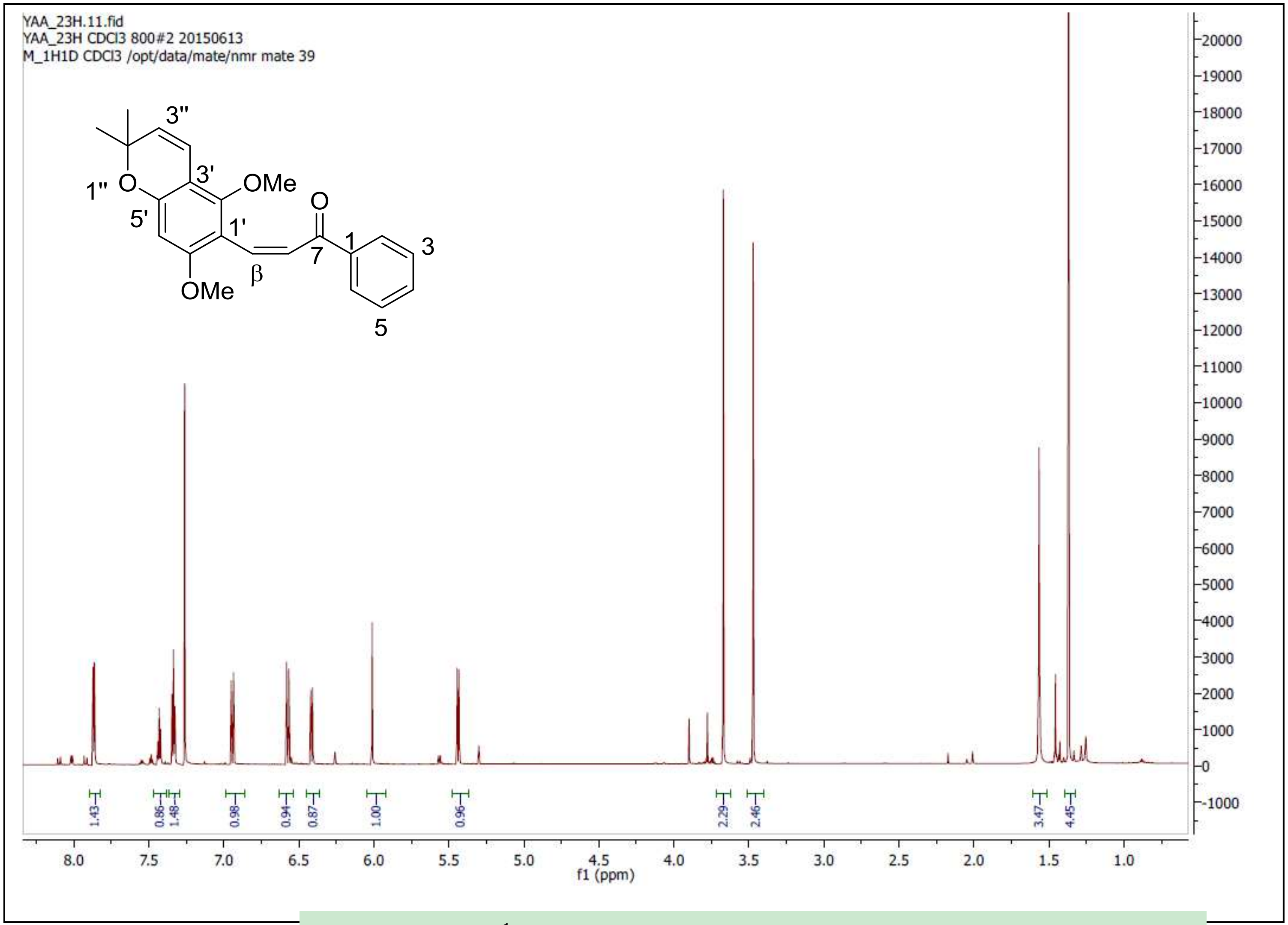

Fig. S9: ^{1}H NMR spectrum of compound **2** (800 MHz; $CDCl_3$)

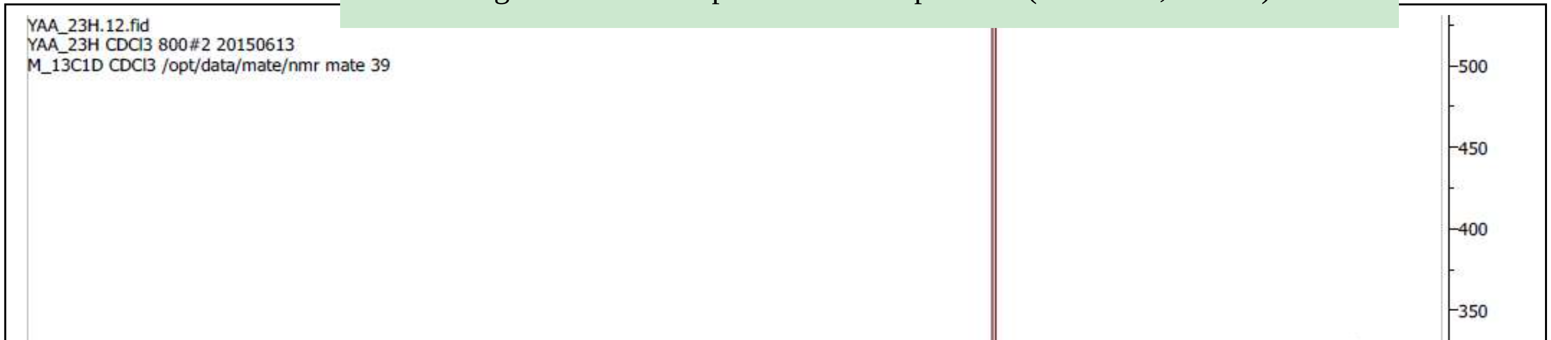

Fig. S10: ^{13}C NMR spectrum of compound **2** (200 MHz; $CDCl_3$)

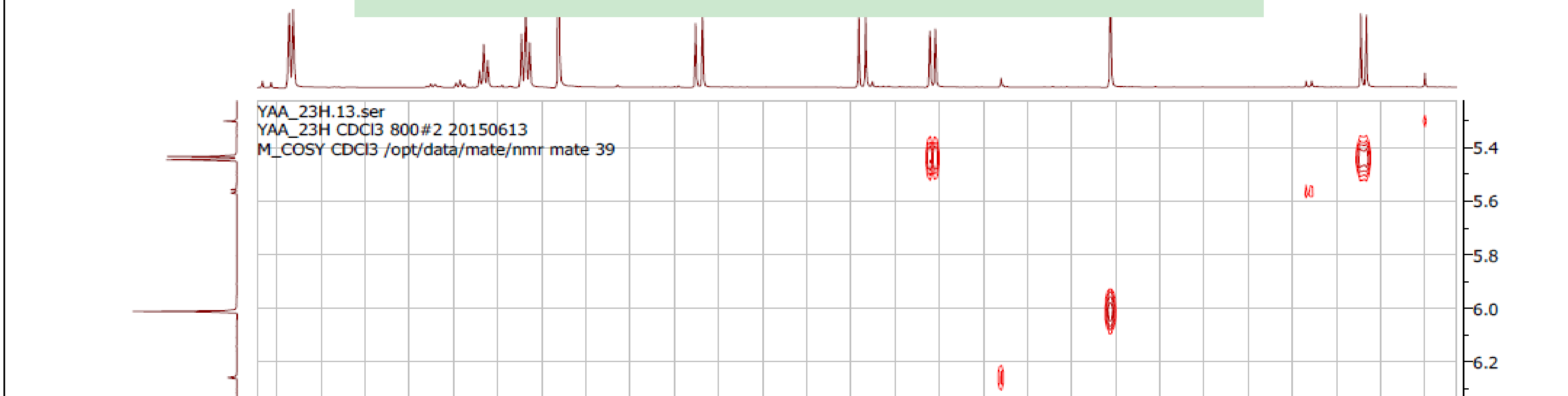

Fig. S11: COSY spectrum of compound **2** (800 MHz; $CDCl_3$).

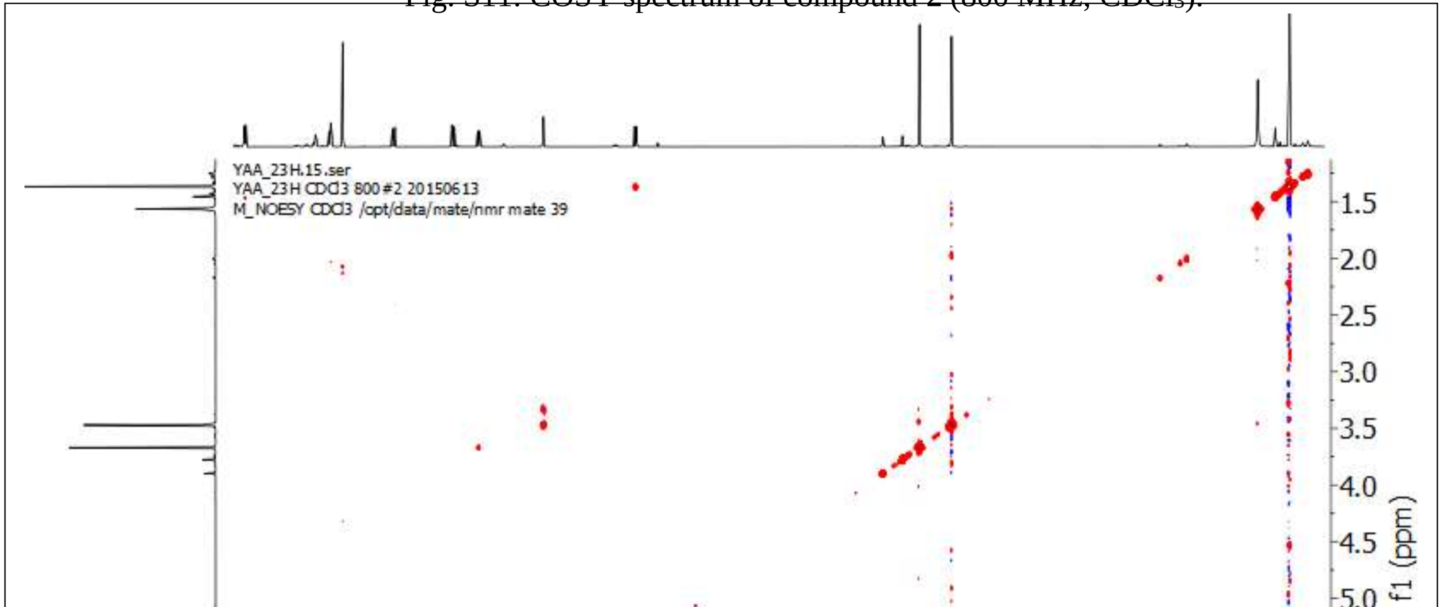

Fig. S12: NOESY spectrum of compound **2** (800 MHz; $CDCl_3$).

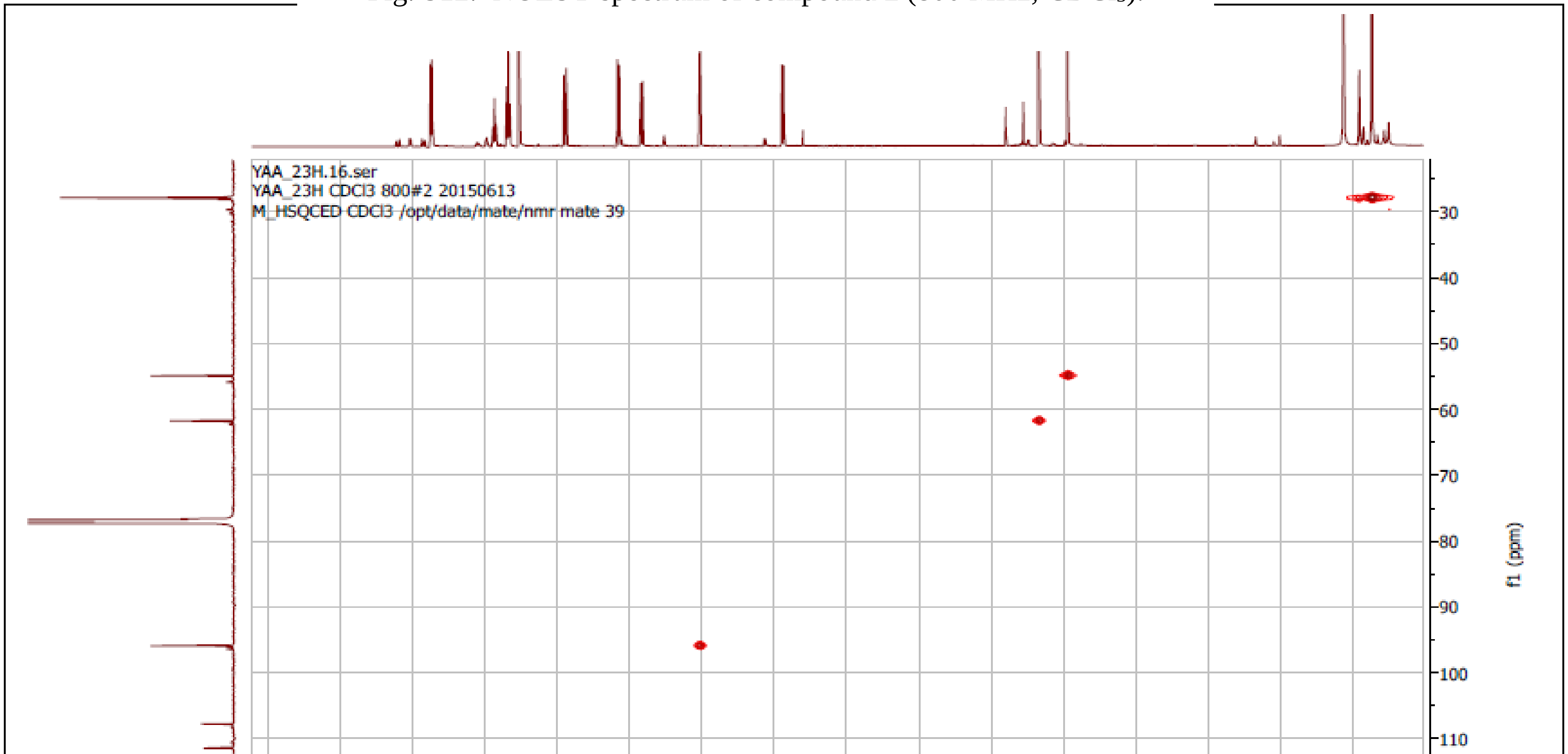

Fig. S13: HSQC spectrum of compound **2** (800 MHz; $CDCl_3$)

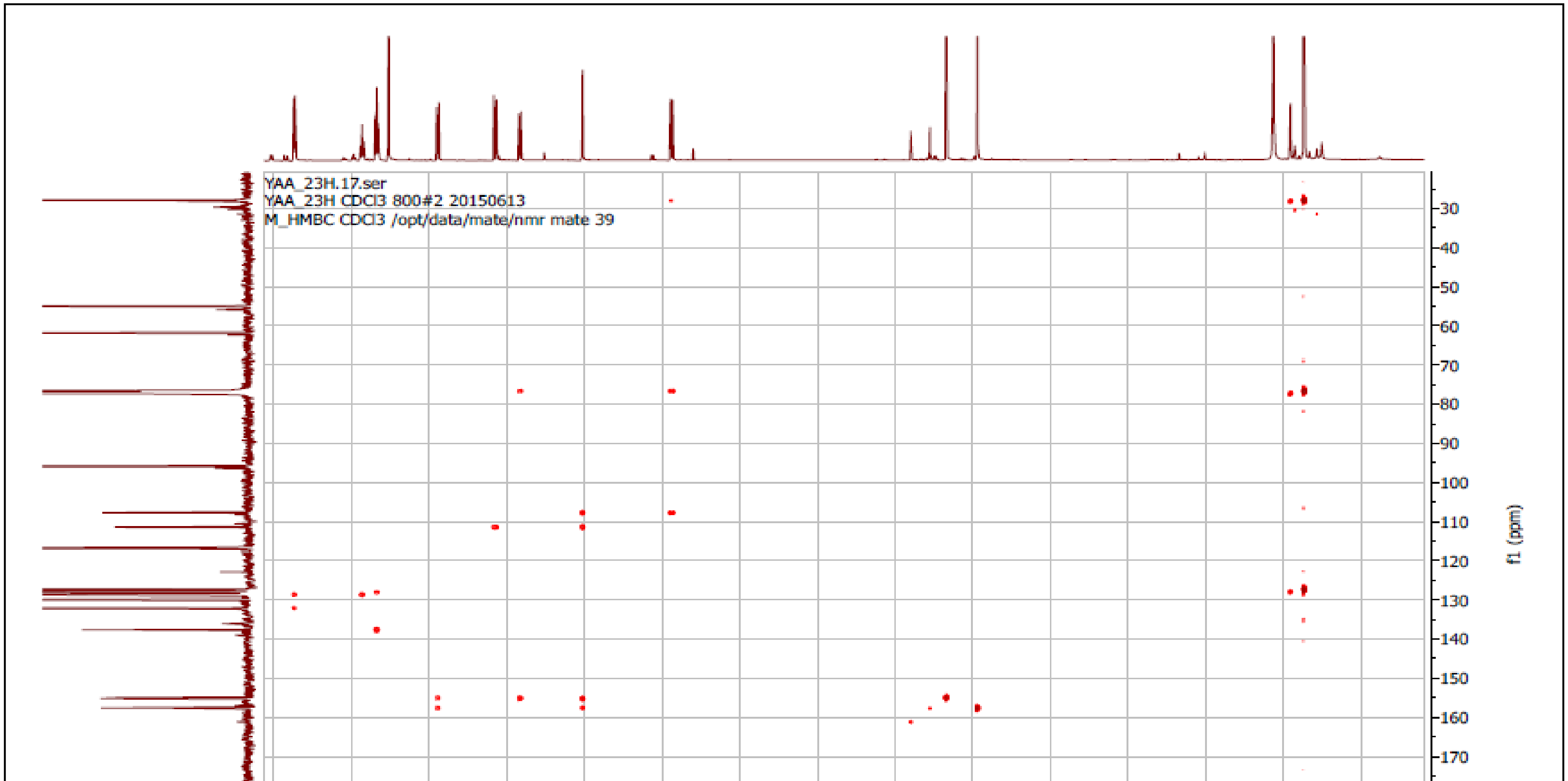

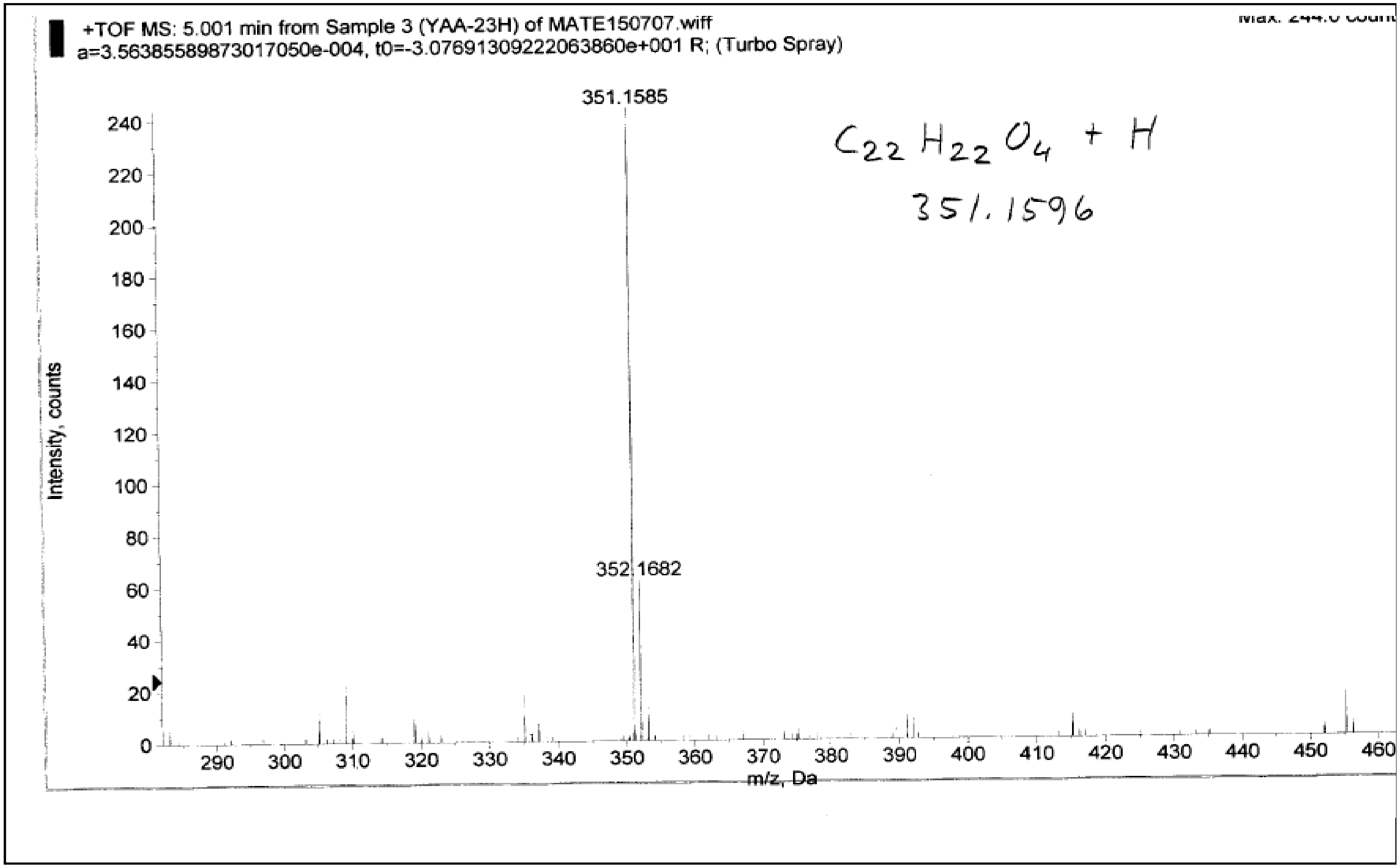
+TOF MS: 5.001 min from Sample 3 (YAA-23H) of MATE150707.wiff
a=3.56385589873017050e-004, t0=-3.07691309222063860e+001 R; (Turbo Spray)
351.1585
352.1682
Intensity, counts
m/z, Da
$C_{22}H_{22}O_4$ + H
351.1596

Fig. S15: HRMS of compound **2**.

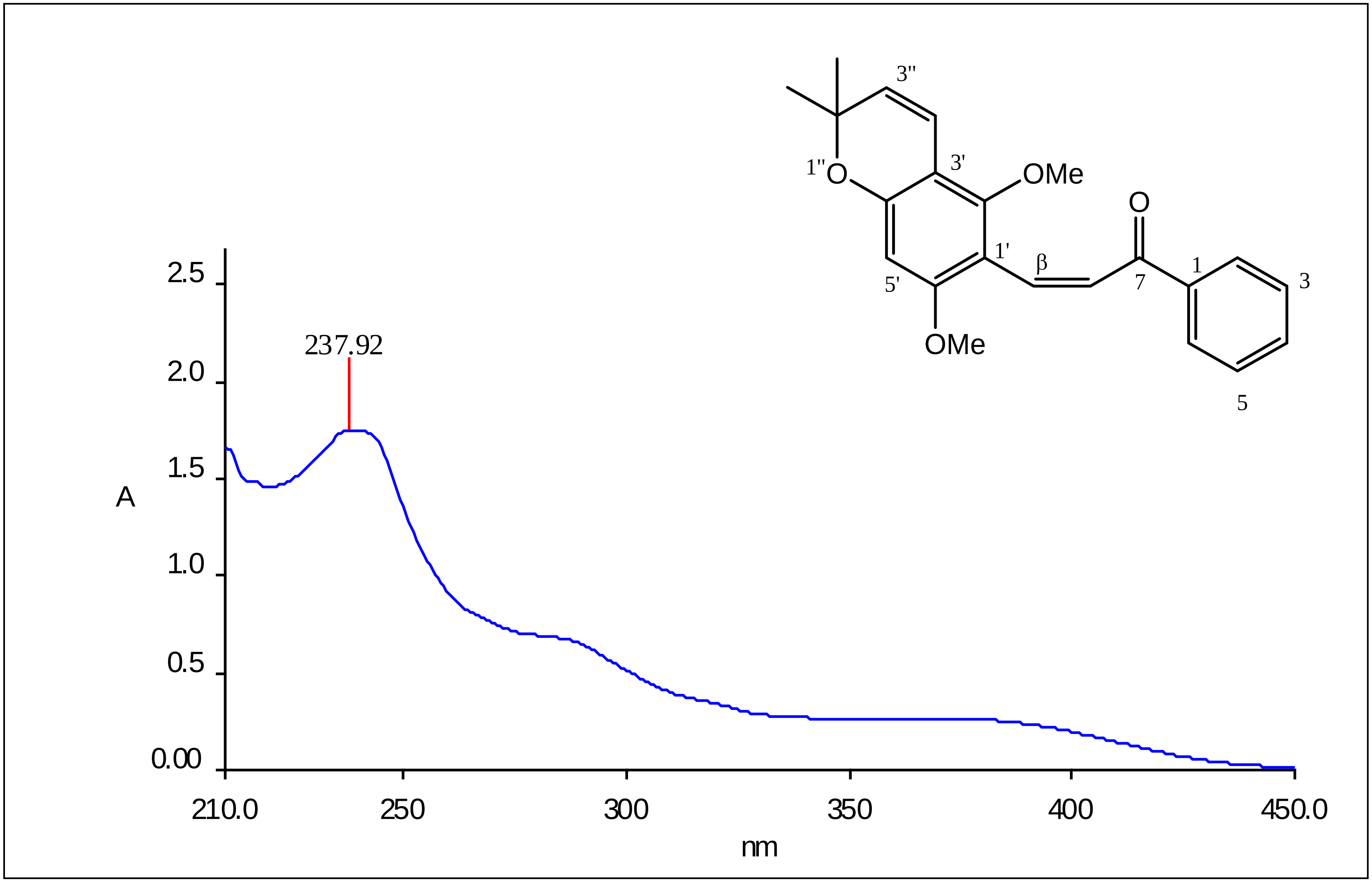

Fig. S16: UV-Vis of compound **2**.

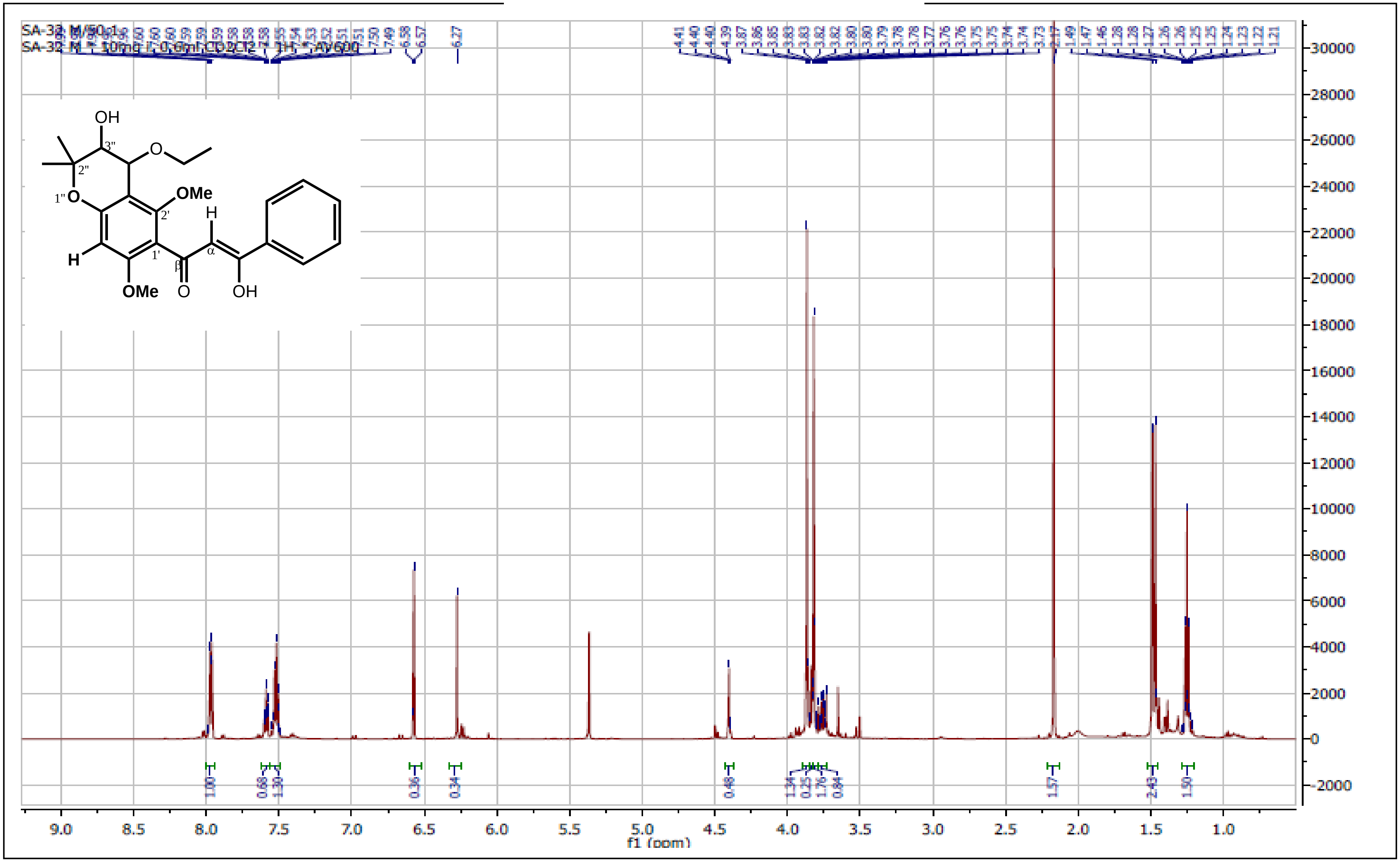

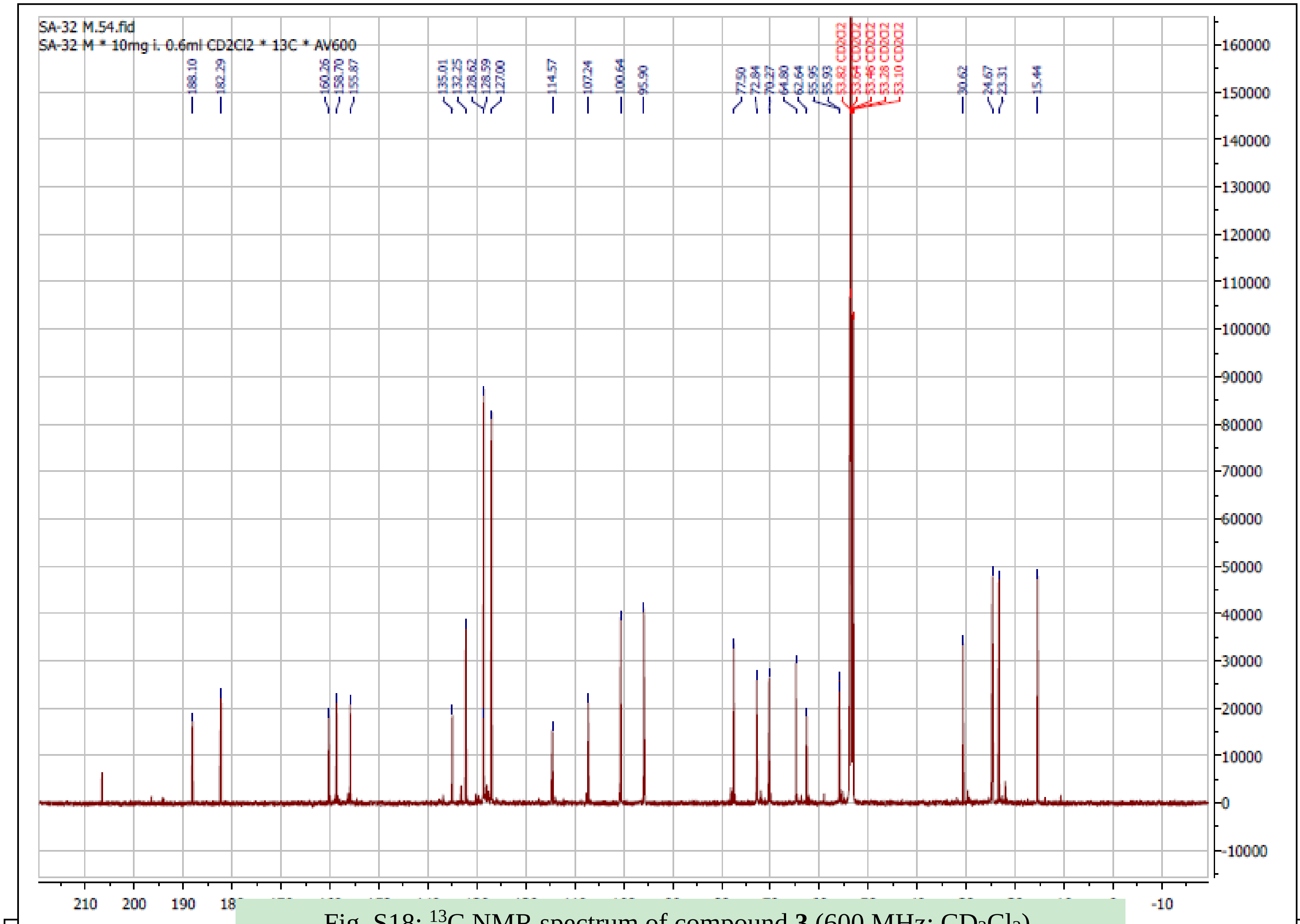

Fig. S18: ^{13}C NMR spectrum of compound **3** (600 MHz; CD_2Cl_2).

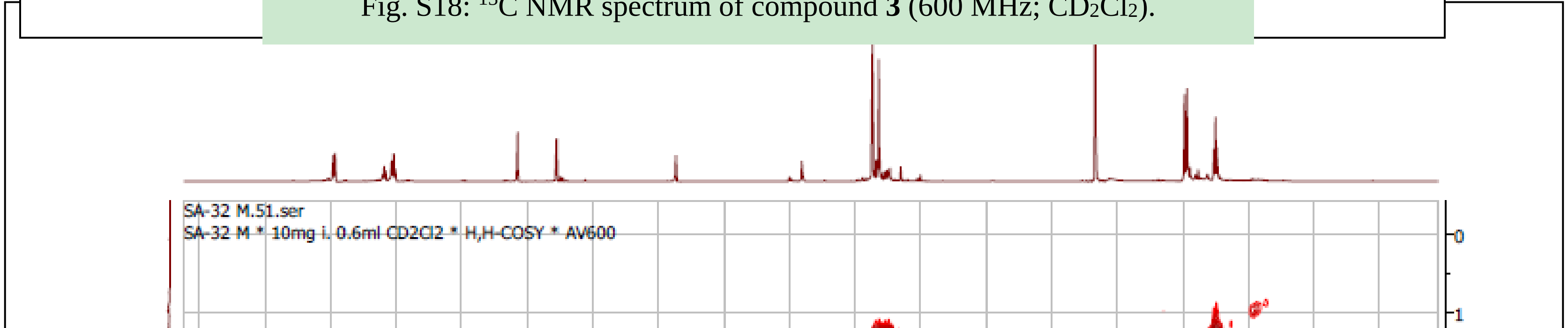

Fig. S19: COSY spectrum of compound **3** (600 MHz; CD_2Cl_2)

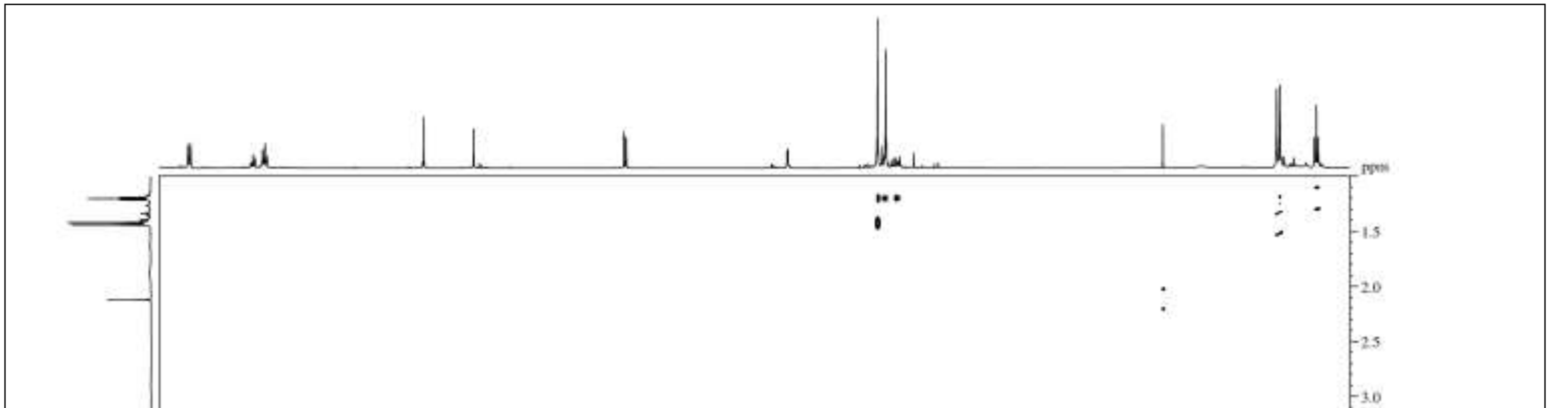

Fig. S20: NOESY spectrum of compound **3** (600 MHz; CD_2Cl_2)

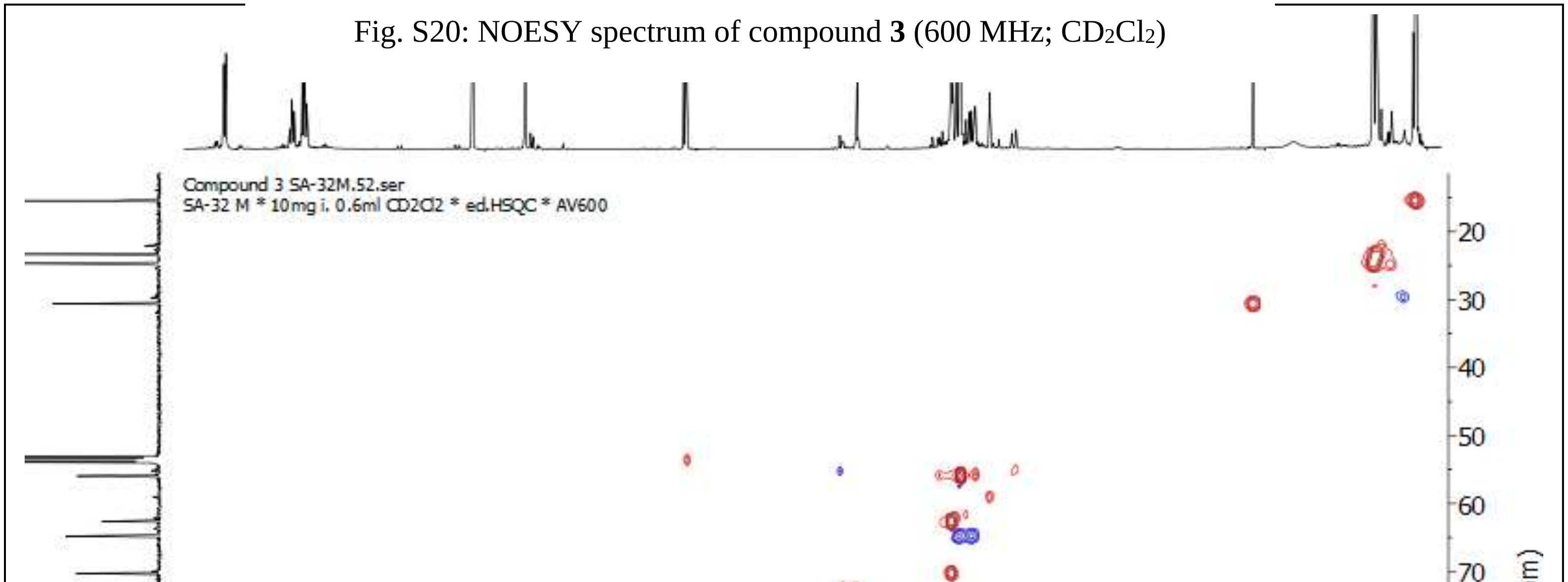

Fig. S21: edited HSQC spectrum of compound **3** (600 MHz; CD_2Cl_2)

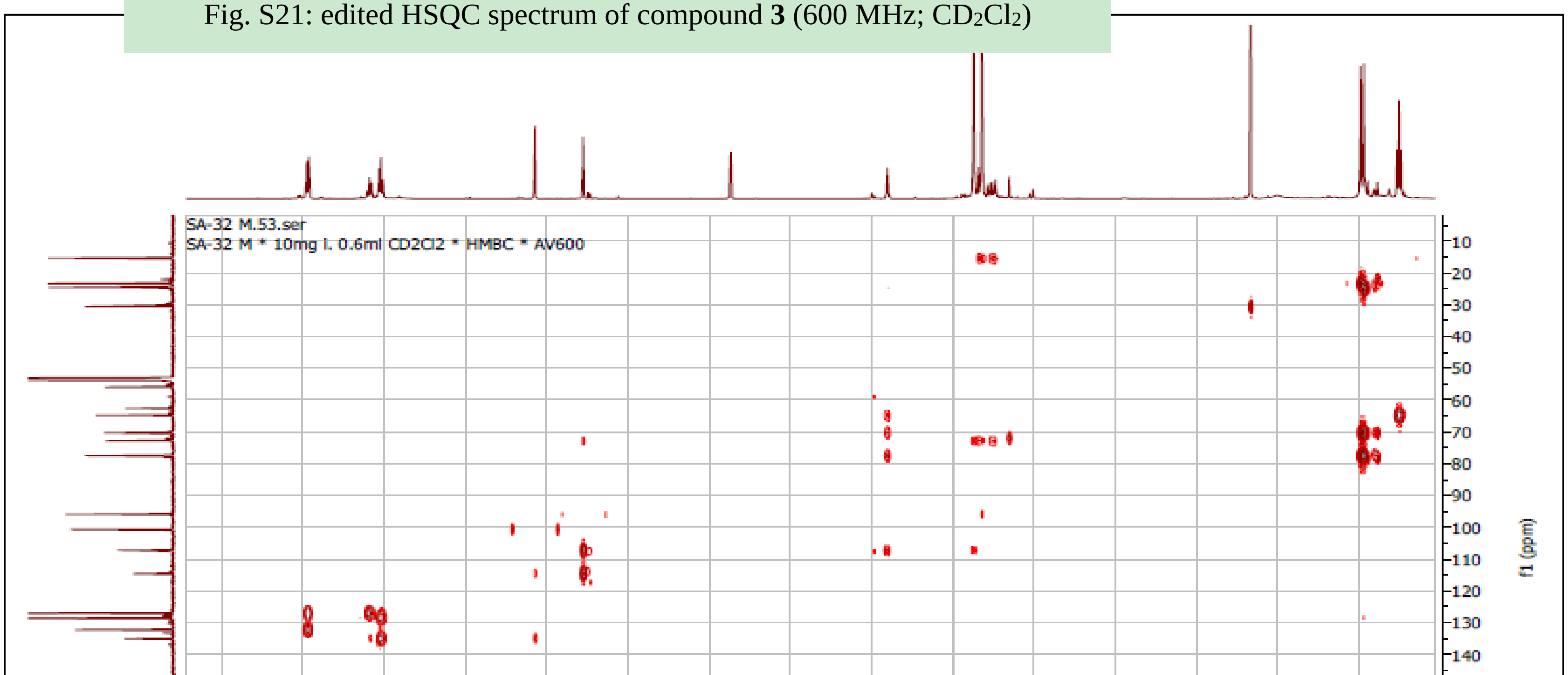

Fig. S22: HMBC spectrum of compound **3** (600 MHz; CD_2Cl_2)

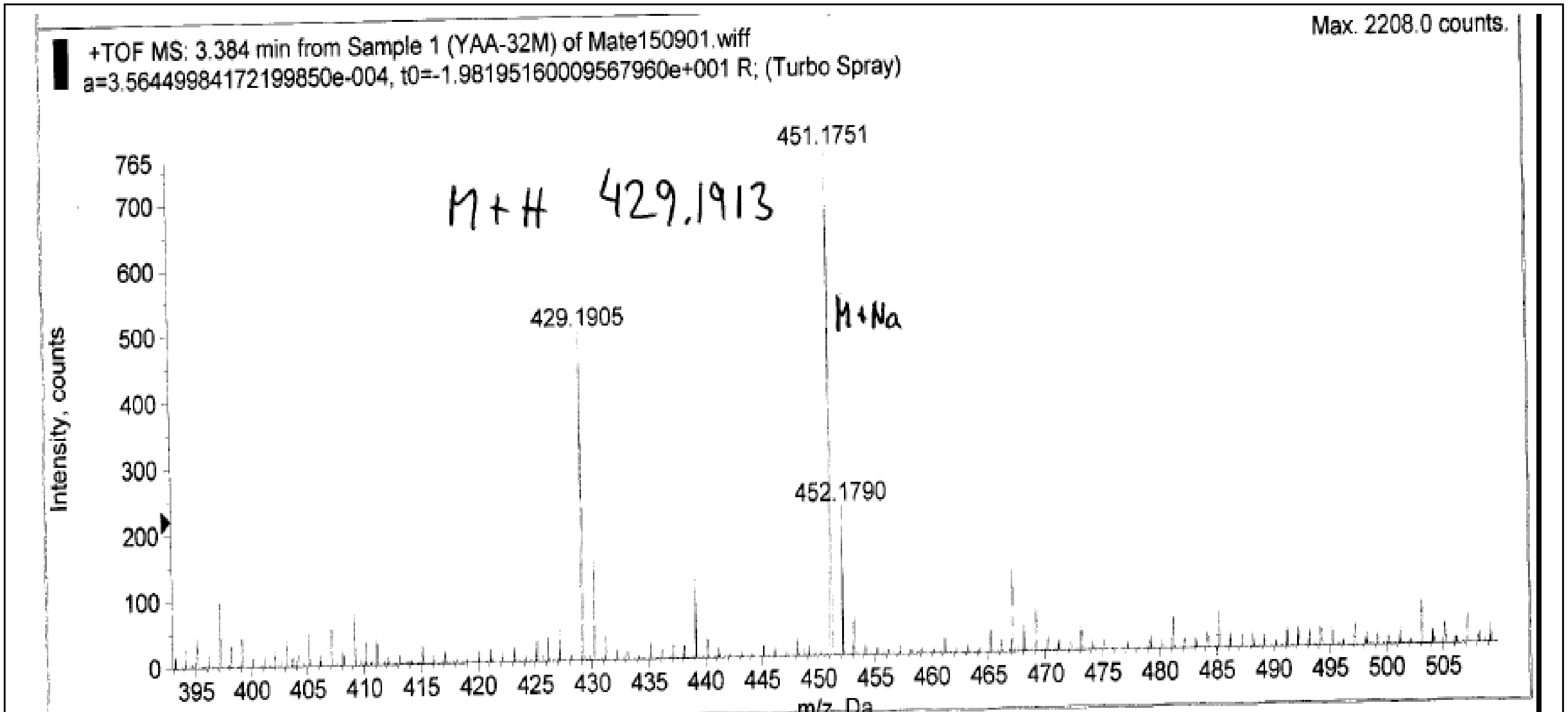

Fig. S23: HRMS of compound **3**

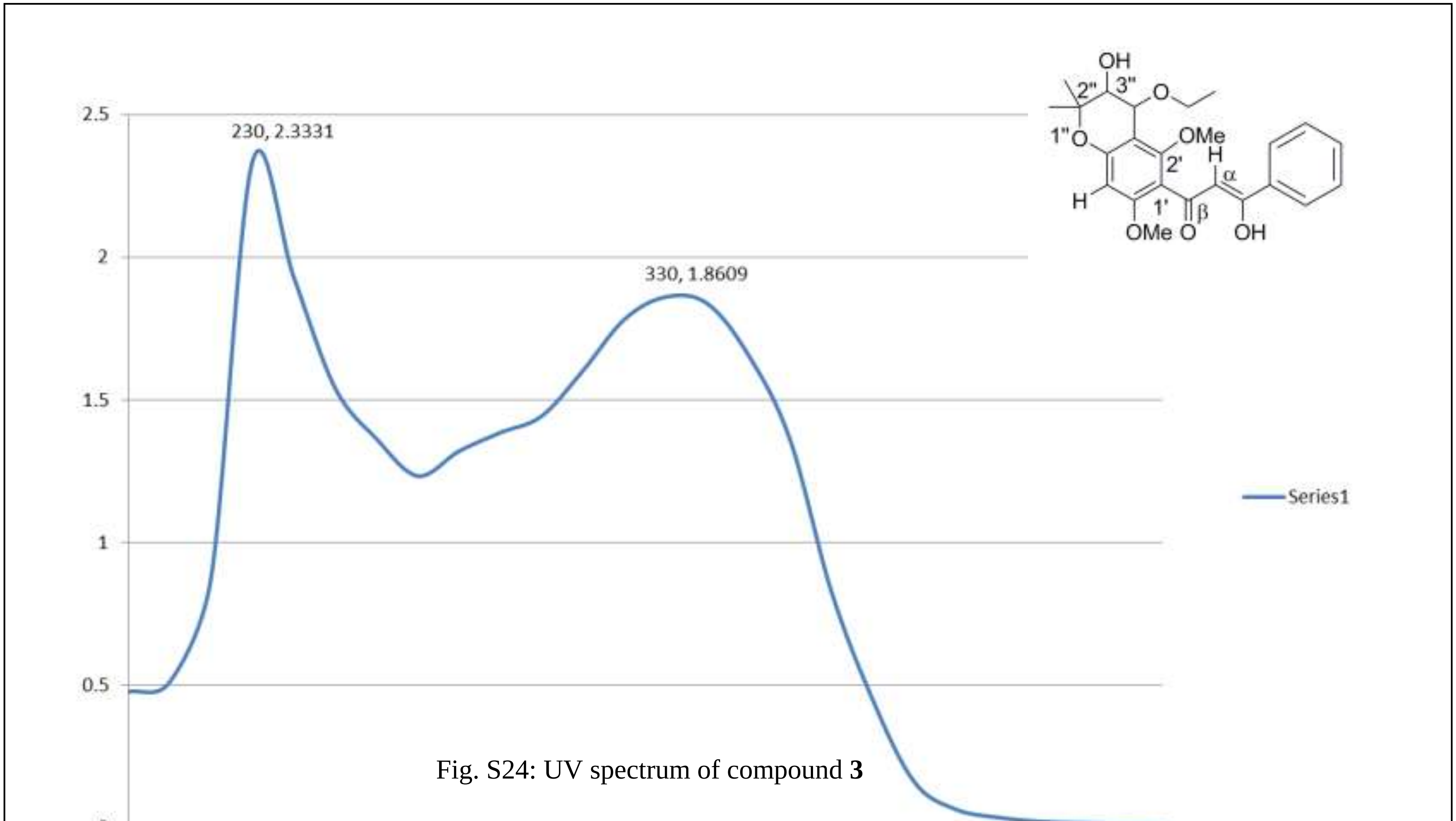

Fig. S24: UV spectrum of compound **3**

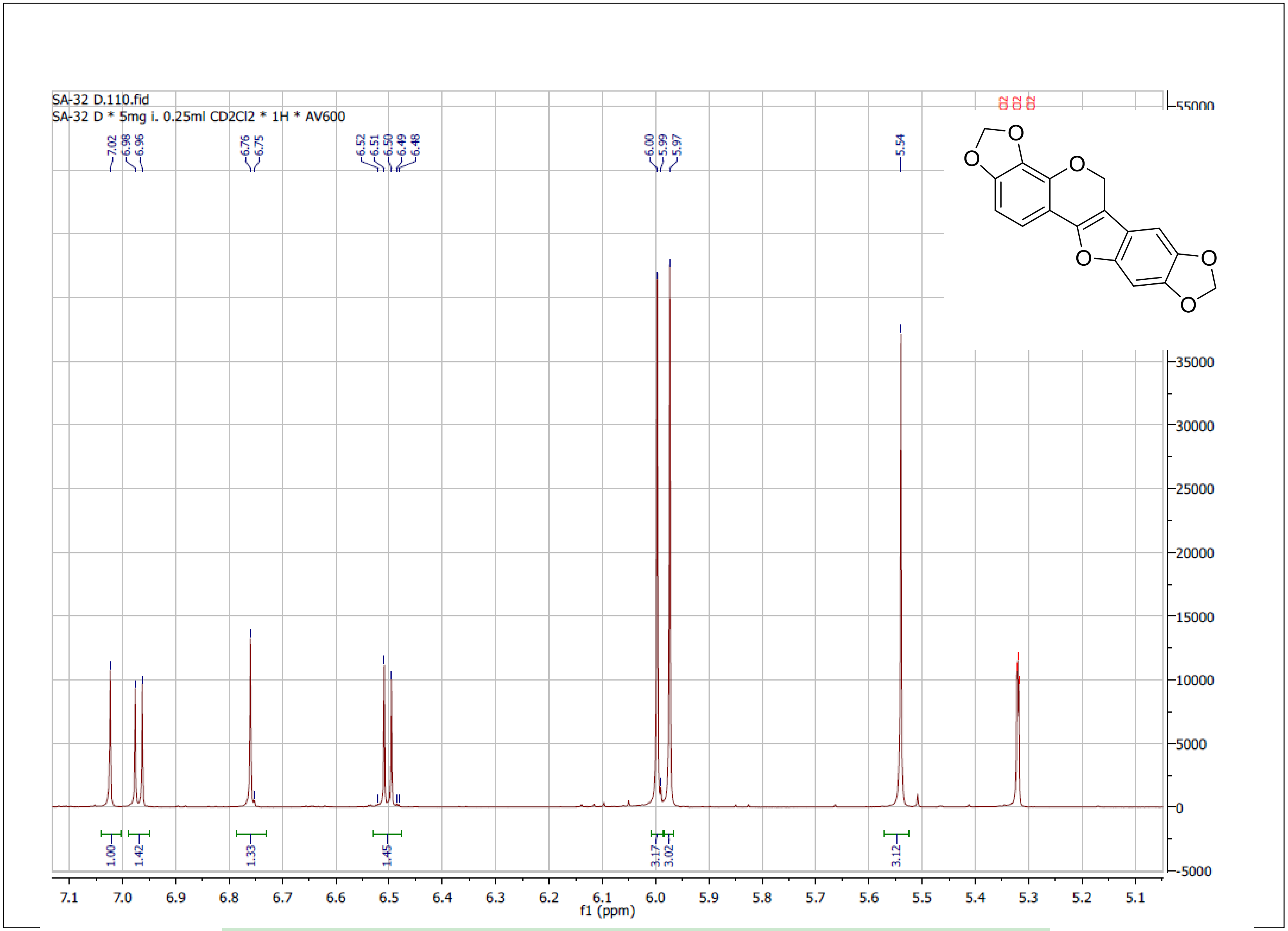

Fig. S25: ^{1}H NMR spectrum of compound **4** (600 MHz; CD_2Cl_2)

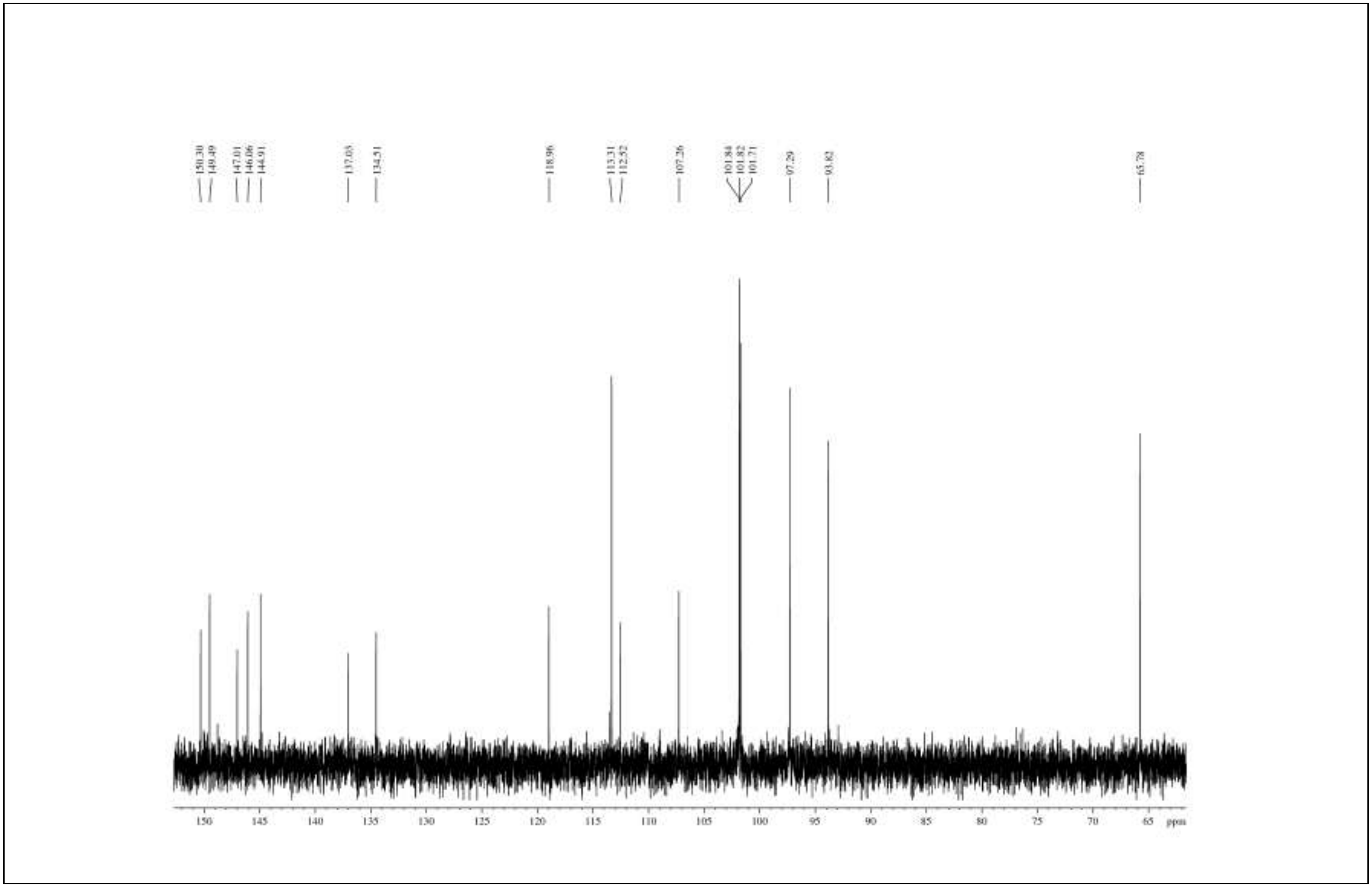

Fig. S26: ^{13}C NMR spectrum of compound **4** (150 MHz; CD_2Cl_2)

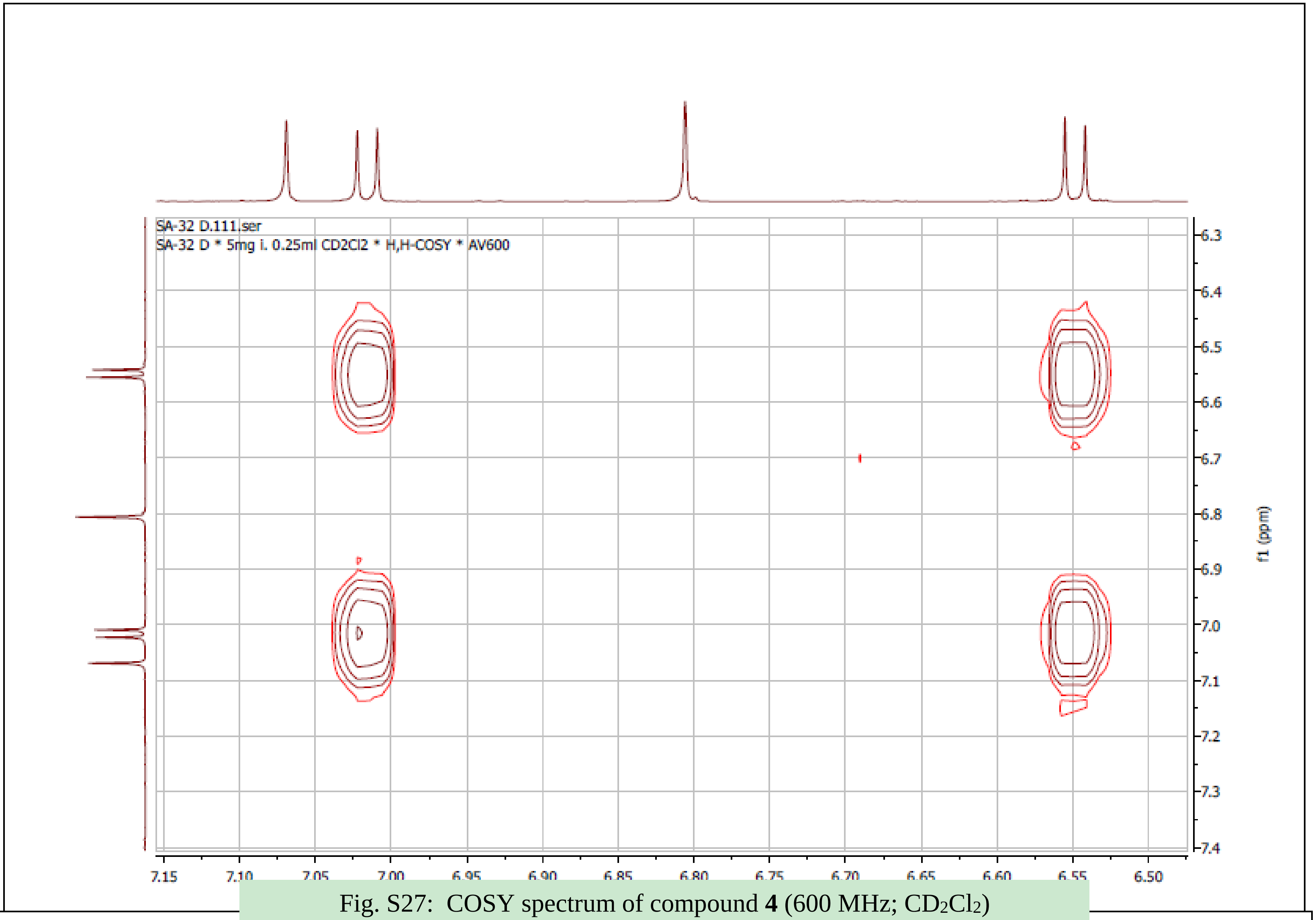

Fig. S27: COSY spectrum of compound **4** (600 MHz; CD_2Cl_2)

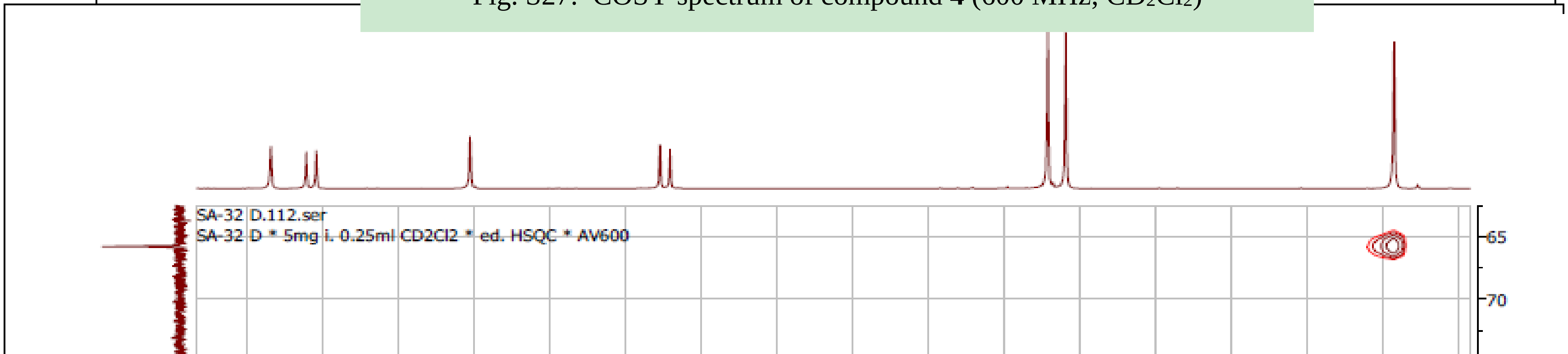

Fig. S28: HSQC spectrum of compound **4** (600 MHz; CD_2Cl_2)

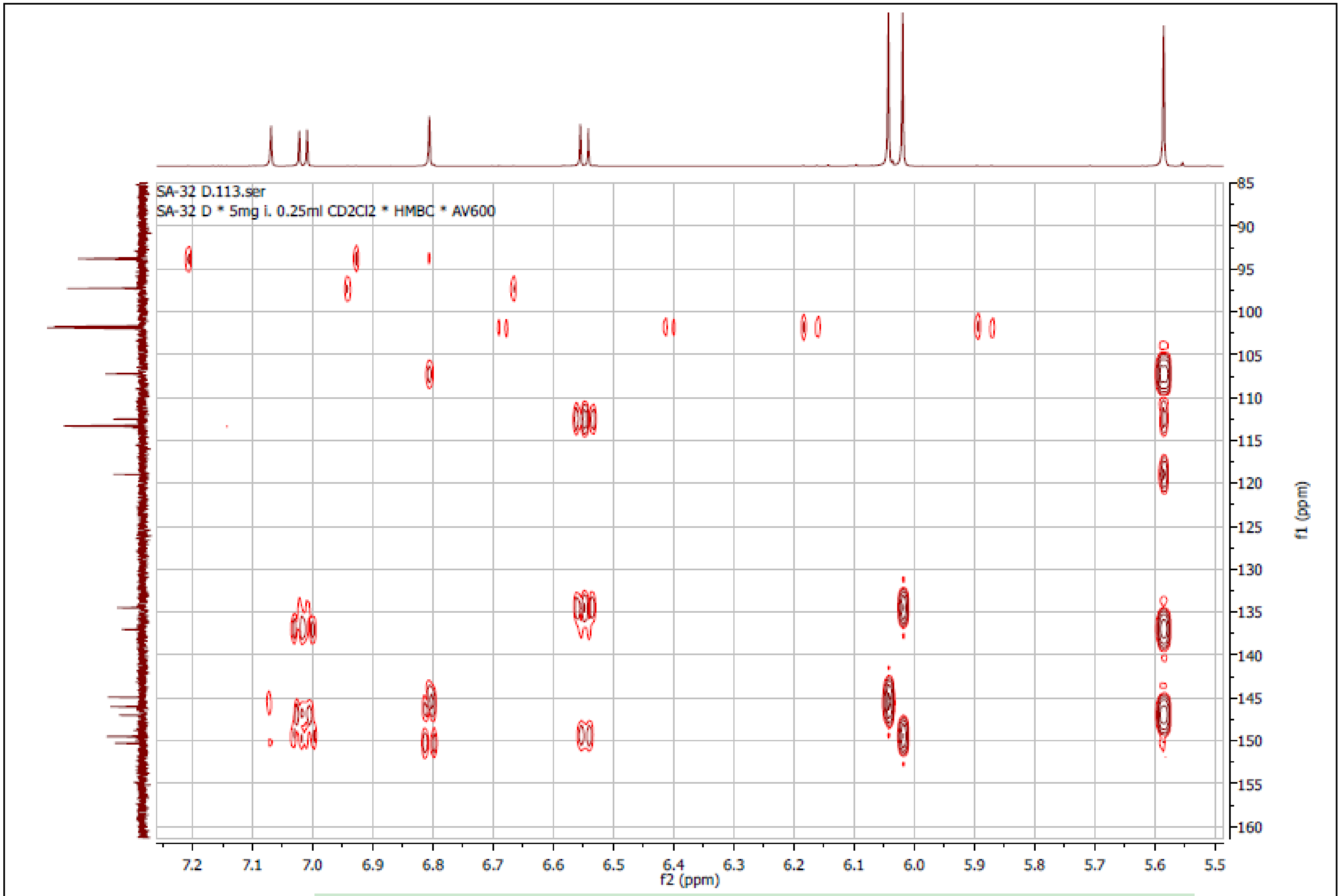

Fig. S29: HMBC spectrum of compound **4** (600 MHz; CD_2Cl_2)

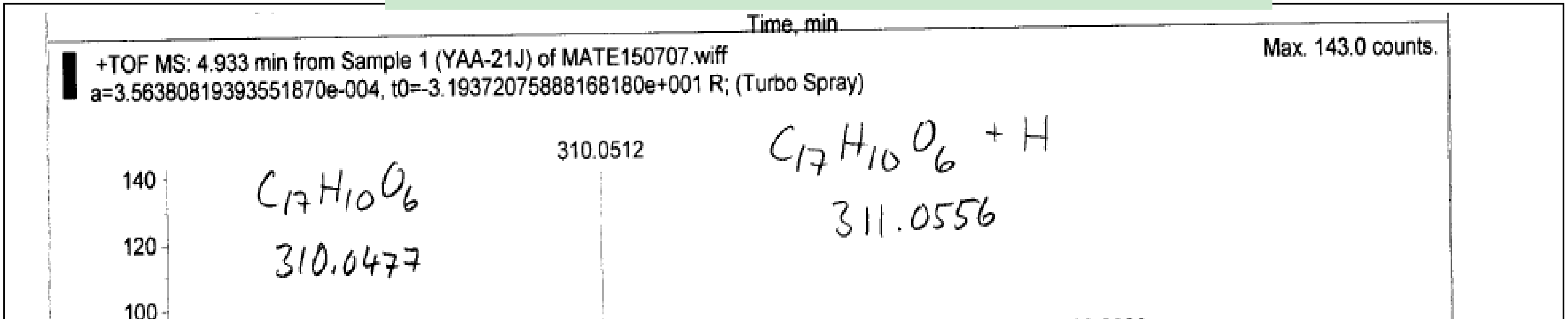

Fig. S30: HRMS of compound **4**

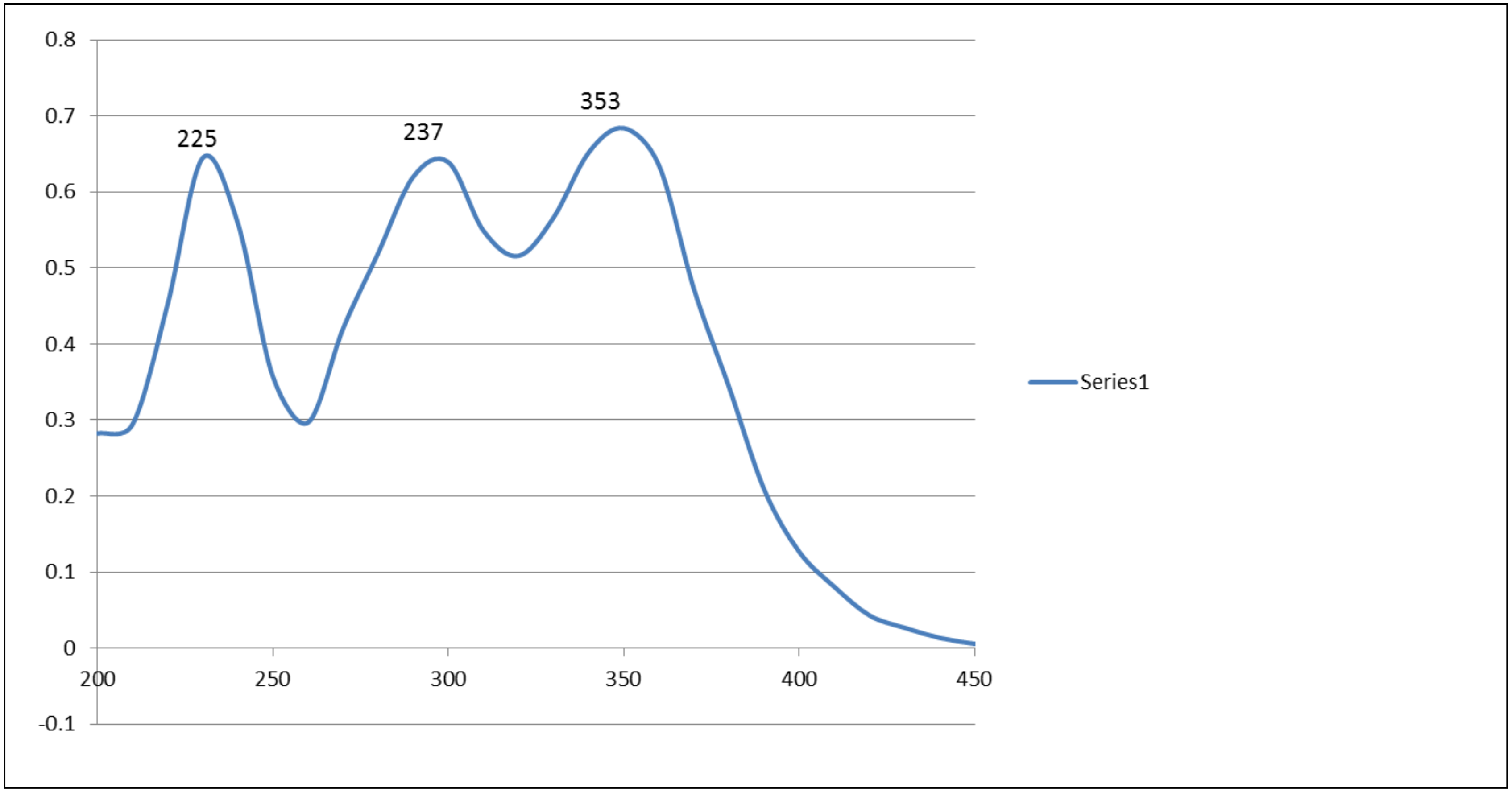

Fig. S31: UV spectrum of compound **4**
